# Metabolomic Comparison of *Saccharomyces cerevisiae* and the Cryotolerant Species *S. bayanus* var. *uvarum* and *S. kudriavzevii* during Wine Fermentation at Low Temperature

**DOI:** 10.1371/journal.pone.0060135

**Published:** 2013-03-20

**Authors:** María López-Malo, Amparo Querol, José Manuel Guillamon

**Affiliations:** 1 Departamento de Biotecnología de los Alimentos, Instituto de Agroquímica y Tecnología de los Alimentos (CSIC), Valencia, Spain; 2 Biotecnologia Enològica, Departament de Bioquímica i Biotecnologia, Facultat d'Enologia, Universitat Rovira i Virgili, Tarragona, Spain; University of Nottingham, United Kingdom

## Abstract

Temperature is one of the most important parameters affecting the length and rate of alcoholic fermentation and final wine quality. Wine produced at low temperature is often considered to have improved sensory qualities. However, there are certain drawbacks to low temperature fermentations such as reduced growth rate, long lag phase, and sluggish or stuck fermentations. To investigate the effects of temperature on commercial wine yeast, we compared its metabolome growing at 12°C and 28°C in a synthetic must. Some species of the *Saccharomyces* genus have shown better adaptation at low temperature than *Saccharomyces cerevisiae*. This is the case of the cryotolerant yeasts *Saccharomyces bayanus* var. *uvarum* and *Saccharomyces kudriavzevii*. In an attempt to detect inter-specific metabolic differences, we characterized the metabolome of these species growing at 12°C, which we compared with the metabolome of *S. cerevisiae* (not well adapted at low temperature) at the same temperature. Our results show that the main differences between the metabolic profiling of *S. cerevisiae* growing at 12°C and 28°C were observed in lipid metabolism and redox homeostasis. Moreover, the global metabolic comparison among the three species revealed that the main differences between the two cryotolerant species and *S. cerevisiae* were in carbohydrate metabolism, mainly fructose metabolism. However, these two species have developed different strategies for cold resistance. *S. bayanus* var. *uvarum* presented elevated shikimate pathway activity, while *S. kudriavzevii* displayed increased NAD^+^ synthesis.

## Introduction

Temperature is one of the main relevant environmental variables that microorganisms have to cope with. The natural environment for the majority of microorganisms, including yeast species, exhibits temporal fluctuations in temperature on scales ranging from daily to seasonal. In addition, temperature is a key factor in some industrial processes involving microorganisms. Low temperatures (10–15°C) are used in wine fermentations to enhance production and to retain flavor volatiles. In this way, white and “rosé” wines of greater aromatic complexity can be achieved [1], [2]. However the optimal growth temperature of the wine yeast *Saccharomyces cerevisiae* is around 32°C [3]. Thus low-temperature fermentation has its disadvantages such an increased lag phase and a reduced growth rate, producing stuck and sluggish fermentations [4]. Therefore the quality of wines produced at low temperature depends on the ability of yeast to adapt to cold.

Despite *S. cerevisiae* being primarily responsible for alcoholic fermentation, other species of the genus *Saccharomyces,* such as *S. bayanus* var. *uvarum,* have been isolated during wine and cider fermentation [5], [6]. Moreover natural interspecific *Saccharomyces* hybrids have been isolated in wine fermentations [7]. These authors identified and characterized new hybrids between *S. cerevisiae* and *S. kudriavzevii*, between *S. cerevisiae* and *S. bayanus*, as well as a triple hybrid *S. bayanus* x *S. cerevisiae* x *S. kudriavzevii*. However *S*. *kudriavzevii* has been isolated only from natural environments and was formally described from decaying leaves in Japan [8]. Recently, Sampaio *et al.* [9] and Lopes *et al.* [10] also isolated new strains in Portugal and Spain, respectively. These two species, *S. bayanus* var. *uvarum* and *S*. *kudriavzevii* are considered the most psychrotrophic species of the *Saccharomyces* genus [3], [11], [12]. Moreover, these two cryotolerant species possess other advantages as compared to *S. cerevisiae* in terms of valuable organoleptic properties, such as greater glycerol production and lower ethanol production [11], [13].

In past years, some attempts have been made to elucidate the cold response in *S. cerevisiae* using a variety of high-throughput methodologies. Some studies have analyzed the genome-wide transcriptional response of *S. cerevisiae* to low temperatures. These studies have mainly focused on cold shock [14], [15], [16], [17]. Schade *et al*. [16] identified two distinct phases during the cold shock response: 1) an early cold response (ECR) occurring within the first 12 h after exposure to low temperature and 2) a late cold response occurring beyond 12 h after exposure to low temperature. ECR-induced genes are implicated in RNA and lipid metabolism, whereas the genes induced during LCR mainly encode the proteins involved in protecting the cell against a variety of stresses. In fact, the LCR response is very similar to the general stress response mediated by transcription factors Msn2p/Msn4p. However, the response type depends on the length of exposure to stressful conditions. Sudden exposure to environmental changes (e.g., cold shock) is likely to trigger a rapid, highly dynamic stress-response (adaptation). Prolonged exposure to non lethal stimuli leads to acclimation; i.e., establishment of a physiological state in which regulatory mechanisms, like gene expression, fully adapt to suboptimal environmental conditions [18]. Tai *et al.* [18] compared their transcriptomic results obtained during cold acclimation in a steady-state chemostat culture with other previous genome-wide transcriptional studies of batch cultures at low temperature, and they found major discrepancies among the low-temperature transcriptome datasets. The authors partially explained these major differences by the cultivation method used in different transcriptome experiments. Although batch cultures are well-suited to study low temperature adaptation dynamics, they are poorly adapted to study prolonged exposure to low temperature. In such cultures, the specific growth rate (µ) is strongly affected by temperature, which makes it impossible to dissect temperature effects on transcription from specific growth rate effects. Two recent chemostat studies [19], [20] also found that the growth rate itself has a strong effect on transcriptional activity. Furthermore, chemostat cultures help to accurately control the specific growth rate, so the concentration of all the metabolites is constant over time, thus providing a good platform to study microbial physiology and gene expression. These genome-wide studies have also tackled the transcriptional response of *S. cerevisiae* [21], *S. kudriavzevii* and their natural hybrids [22] in enological conditions.

In addition to transcriptomic, there are the other so-called “omics” currently available, such proteomics and metabolomics. In our group, we previously studied the proteome changes of a commercial wine yeast strain during the first hours of fermentation at low temperature [23]. However, there is a limited body of knowledge on the application of metabolomics to the winemaking process, and it has never been applied to understand yeast cold adaptation under fermentation conditions. The metabolome comprises the complete set of metabolites, these being the non-genetically encoded substrates, intermediates and products of the metabolic pathways associated with a cell. By representing integrative information across multiple functional levels and by linking DNA encoded processes with the environment, the metabolome offers a window to the map core attributes responsible for different phenotypes [24]. Few studies have applied metabolomics to the winemaking process. Skogerson *et al.* [25] determined the metabolite profiles of white wines belonging to different grape varieties. As far as we know, comprehensible metabolic profiles have not yet been determined in yeast growing under conditions that mimic industrial fermentations.

The aim of this study is to improve the feasibility of low-temperature wine fermentation by identifying biomarkers and metabolic adaptations at low temperature in the industrial wine yeast strain QA23, and to detect differential metabolites that distinguish two cryotolerants *S. bayanus* var. *uvarum* and *S. kudriavzevii* species from *S. cerevisiae,* whose growth potential is limited at this temperature. To achieve these objectives, we analyzed the global metabolic profiling of *S. cerevisiae* growing in a steady-state chemostat at 12°C and 28°C at the same growth rate. Thus, for a better understanding of the low-temperature adaptation of *S. bayanus* var. *uvarum* and *S. kudriavzevii*, we characterized the metabolome of these species which we compared with the metabolome of commercial wine yeast QA23 at 12°C.

## Materials and Methods

### Yeast strains and culture conditions

A commercial *S. cerevisiae* (*Sc*) wine strain (QA23, Lallemand S.A., Canada), a *S. bayanus* var. *uvarum* (*Su*) strain (CECT12600) and a *S. kudriavzevii* strain (*Sk*) (CR85) [10] were used in this work. The *Sc* strain was grown at a dilution rate (D) of 0.04 h^−1^ at 12°C and 28°C in 2 L chemostat (Biostat ® B, Braun Biotech International, Sartorius Group, Germany) with a working volume of 0.75 L. The *Su* and *Sk* strains were grown under the same conditions, but only at 12°C. A temperature probe connected to a cryostat controlled the cultures grown at 12°C.

Cultures were grown in the synthetic grape must (SM) derived from that described by Bely *et al.* [26]. The medium composition included 200 g L^−1^ of sugars (100 g L^−1^ glucose + 100 g L^−1^ fructose), 6 g L^−1^ malic acid, 6 g L^−1^ citric acid, 1.7 g L^−1^ YNB without ammonium and amino acids, anaerobic factors (15 mg L^−1^ ergosterol, 5 mg L^−1^ sodium oleate and 0.5 mL L^−1^ tween 80) and 60 mg L^−1^ potassium disulfite. The assimilable nitrogen source used was 300 mg N L^−1^ (120 mg N L^−1^ as ammonium and 180 mg N L^−1^ in an amino acid form). pH was measured online and kept constant at 3.6 by the automatic addition of 2 M NaOH and 1 M HCl. The stirrer was set at 100 rpm. Biomass and extracellular metabolites were constant for at least five volume changes before sampling. Sampling of cells from continuous cultures was carried out following the “general sample preparation recommendations for metabolon studies” of Metabolon, Inc. (Durham, NC, USA). When the steady state was reached, a volume of approximately 30 units of OD_600_ was centrifuged at 1000 g for 3 min at 4°C. After removing the supernatant, the cell suspension was washed with PBS, transferred to a 1.5–2.0 ml microcentrifuge tube and centrifuged again under the same conditions. The pellet was flash-frozen with liquid nitrogen and stored at −80°C.

### HPLC analysis

Glucose, fructose, glycerol and ethanol were analyzed in all the supernatant samples. Analytical HPLC was carried out in a Surveyor Plus Chromatograph (Thermo Fisher Scientific, Waltham, MA) equipped with a refraction index detector, autosampler and a UV-Visible detector. Prior to injection, samples were centrifuged at 13300 rpm for 5 min, and samples were diluted 10-fold and filtered through 0.22-µm pore size nylon filters (Micron Analitica, Spain). A total volume of 25 µL was injected into a HyperREZ XP Carbohydrate H+8 µm column (Thermo Fisher Scientific) assembled to its correspondent guard. The mobile phase used was 1.5 mM H_2_SO_4_ with a flux of 0.6 mL min^−1^ and a column temperature of 50°C. The concentration of each was calculated using external standards. Each sample was analyzed in duplicate.

### Nitrogen content analysis

Ammonia concentration was measured with a kit following an enzymatic method (Roche Applied Science, Germany). The concentration of free amino acid nitrogen was determined by the σ-phthaldehyde/N-acetyl-L-cysteine spectrophotometric assay (NOPA) procedure [27]. The results were expressed as mg nitrogen mL^−1^.

### Metabolic Profiling

Frozen samples of cells, OD of approximately 30 units, were submitted to Metabolon, Inc. (Durham, NC, USA) for sample extraction and analysis. The sample preparation process was carried out using the automated MicroLab STAR® system from the Hamilton Company. Sample preparation was conducted using a proprietary series of organic and aqueous extractions. The resulting extract was divided into two fractions; one for analysis by liquid chromatography/mass spectrometry (LC/MS) and one for analysis by gas chromatography/mass spectrometry (GC/MS). Samples were placed briefly in a TurboVap® (Zymark) to remove the organic solvent. The LC/MS extracts reconstituted under acidic conditions were gradient-eluted using water and methanol, both containing 0.1% formic acid, while basic extracts, which also used water/methanol, contained 6.5 mM ammonium bicarbonate. The samples destined for the GC/MS analysis were re-dried under vacuum desiccation for a minimum of 24 h prior to being derivatized under dried nitrogen using bistrimethyl-silyl-triflouroacetamide (BSTFA).

The LC/MS portion of the platform was based on a Waters ACQUITY UPLC and a Thermo-Finnigan LTQ mass spectrometer, which consisted of electrospray ionization (ISI) and a linear ion-trap (LIT) mass analyzer. The derivatized samples for GC/MS were analyzed in a Thermo-Finnigan Trace DSQ fast-scanning single-quadripole mass spectrometer using electron impact ionization. The GC column was 5% phenyl and the temperature ramp went from 40°C to 300°C in a 16-minute period. Accurate mass determination and MS/MS fragmentation (LC/MS), (LC/MS/MS) were carried out in a Thermo-Finnigan LTQ-FT mass spectrometer, which had an LIT front end and a fourier transform ion cyclotron resonance (FT-ICR) mass spectrometer backend.

Compounds were identified by comparison to Metabolon's library entries of purified standards or recurrent unknown entities. Data were normalized to the correct variation resulting from instrument inter-day tuning differences. Raw area counts for each compound were corrected in run-day blocks by registering the medians to equal one and by normalizing each data point proportionately.

### Statistical data processing

The metabolic data are the result of five replicates for each fermentation (temperature and strains). Significant differences between the supernatants of *Sk*, *Su* and *Sc* at both temperatures and the other two species were determined by *t*-tests. The statistical level of significance was set at *P*≤0.05.

For the metabolic profiling data, two types of statistical analysis were performed. Following log transformation and imputation with minimum observed values for each compound, Welch's two sample t-tests were used to identify the metabolites that differed significantly between the experimental groups. An estimate of the false discovery rate (q-value) was calculated to take into account the multiple comparisons that normally occur in metabolomic-based studies. A low q-value (q<0.10) indicates high confidence in a result. For classification purposes, we mainly used random forest analyses. Random forests create a set of classification trees based on the continual sampling of experimental units and compounds. Statistical analyses were performed with the R program.

Principal component analysis (PCA) was done using the *vegan* package (the *rda* function) of the R v.2.15 statistical software [28].

## Results

### Global biochemical profiles comparison among the three species

This comparative biochemical study aimed to characterize the metabolic adaptations defining *S. cerevisiae* grown at a near-optimal temperature (i.e., 28°C) versus a non optimal temperature (i.e., 12°C), and to compare and contrast its profile to *Saccharomyces* genus members (*S. bayanus* var. *uvarum* and *S. kudriavzevii*) with greater tolerance at low temperature.

Our experimental design was based on continuous-culture fermentations. This system offers a stable and controlled environment for cells by maintaining constant biomass and concentrations of nutrients and products [29], thus making the comparison between fermentation conditions and strains more feasible. All the cultures were grown at the same dilution rate (D), which corresponded to the maximum D of the control condition (*Sc* at 12°C). When the steady state was reached (after five volume changes), the sampling of supernatants and cells was done. Table 1 shows the physiological data of the three *Saccharomyces* strains and the concentration of the main compounds of oenological interest in the supernatant in the steady state. As seen, few sugars were consumed from the SM fed to the continuous cultures, and *Sc* at 28°C was the strain which more consumed sugars. Intriguingly, cryotolerant strains *Su* and *Sk* consumed less than 10% of the initial sugar content. These parameters clearly indicate that the metabolic study fitted the first wine fermentation stages (initial exponential phase). In accordance with this fermentation stage, residual nitrogen (both ammonium and amino acids) was hardly consumed, with high concentrations in the supernatant. The high concentrations of sugars and nitrogen indicated that these chemostat cultures were not carbon-limited or nitrogen-limited. As the steady state was reached in all the cultures, another nutrient, or group of nutrients, must limit growth. The physiological data showed remarkable differences between *Sc* and the cryotolerant species *Su* and *Sk*. Regardless of the growth temperature, the sugar consumption rate and the glycerol and ethanol production rate were higher for *Sc* than for *Su* and *Sk*. Conversely, *Su* and *Sk* presented higher biomass yields than *Sc*. Likewise, the cultures of *Sc* showed no significant differences in biomass yields, in the glucose and ammonium consumption rates, and in the ethanol production rate between both temperatures. Conversely, *Sc* at 12°C displayed greater amino acid consumption and lower glycerol production rates than *Sc* at 28°C.

**Table 1:** Physiological characteristics of *Saccharomyces* strains and extracellular metabolites during the steady state of continuous cultures.

	*Sc* 28°C	*Sc* 12°C	*Su*	*Sk*
Extracellular metabolites				
Glucose (g L^−1^)	72.31±1.73*	78.89±1.02	89.09±1.14*	92.02±1.01*
Fructose (g L^−1^)	83.88±2.00	83.53±1.40	93.18±1.06*	95.08±1.33*
Amino acids (mg NL^−1^)	145.76±8.55*	80.19±4.00	150.73±2.91*	148.55±4.07*
Ammonium (mg L^−1^)	90.71±5.95*	55.18±2.93	105.44±6.03*	121.94±3.29
Glycerol (g L^−1^)	4.62±0.17*	1.60±0.09	1.17± 0.09*	0.91±0.04*
Ethanol (g L^−1^)	19.97±0.45*	13.98±0.78	6.38±0.54*	4.76±0.12*
Physiological data				
Biomass (g DW L^−1^)	1.55± 0.07*	1.15±0.07	0.93±0.11	0.77±0.06*
Y_hexoses_-X (g_DW_.g_hexoses_^−1^)	0.0 4±0.00	0.03±0.00	0.06±0.0*	0.06±0.01*
q_glucose_ (g_glucose_.g DW ^−1^. h^−1^)	−0.71±0.05	−0.73±0.04	−0.44±0.02*	−0.42±0.06*
q_fructose_(g_fructose_.g DW ^−1^. h^−1^)	−0.42±0.05*	−0.57±0.05	−0.26±0.02*	−0.26±0.06*
q_aa_ (mg _Naa_.g DW ^−1^. h^−1^)	−0.88±0.22*	−3.47±0.14	−1.3±0.02*	−1.68±0.22*
q_NH4_ (mg _NH4_.g DW ^−1^. h^−1^)	−1.67±0.08	−1.16±0.37	−0.62±0.32	0.00± 0.00*
q_glycerol_(g _glycerol_.g DW ^−1^. h^−1^)	0.12±0.00*	0.06±0.00	0.05±0.00*	0.05±0.00*
q_ethanol_(g _ethanol_.g DW ^−1^. h^−1^)	0.52±0.01	0.49±0.03	0.28±0.03*	0.25±0.00*

* Significant differences (p value≤0.05) compared to the control condition (*Sc* 12°C).

The comparison of the global metabolic profiles of the control condition *Sc*−12°C versus the other continuous-cultures (*Sc*−28°C, *Su*−12°C and *Sk*-12°C) resulted in 295 molecules identified from a total of 1700 molecules contained within the Metabolon library ( Table S1). These compounds included a wide variety of metabolic classes: amino acids, peptides, carbohydrates, energy metabolisms, lipids, nucleotides, cofactors, vitamins and xenobiotics.

**Figure 1:**
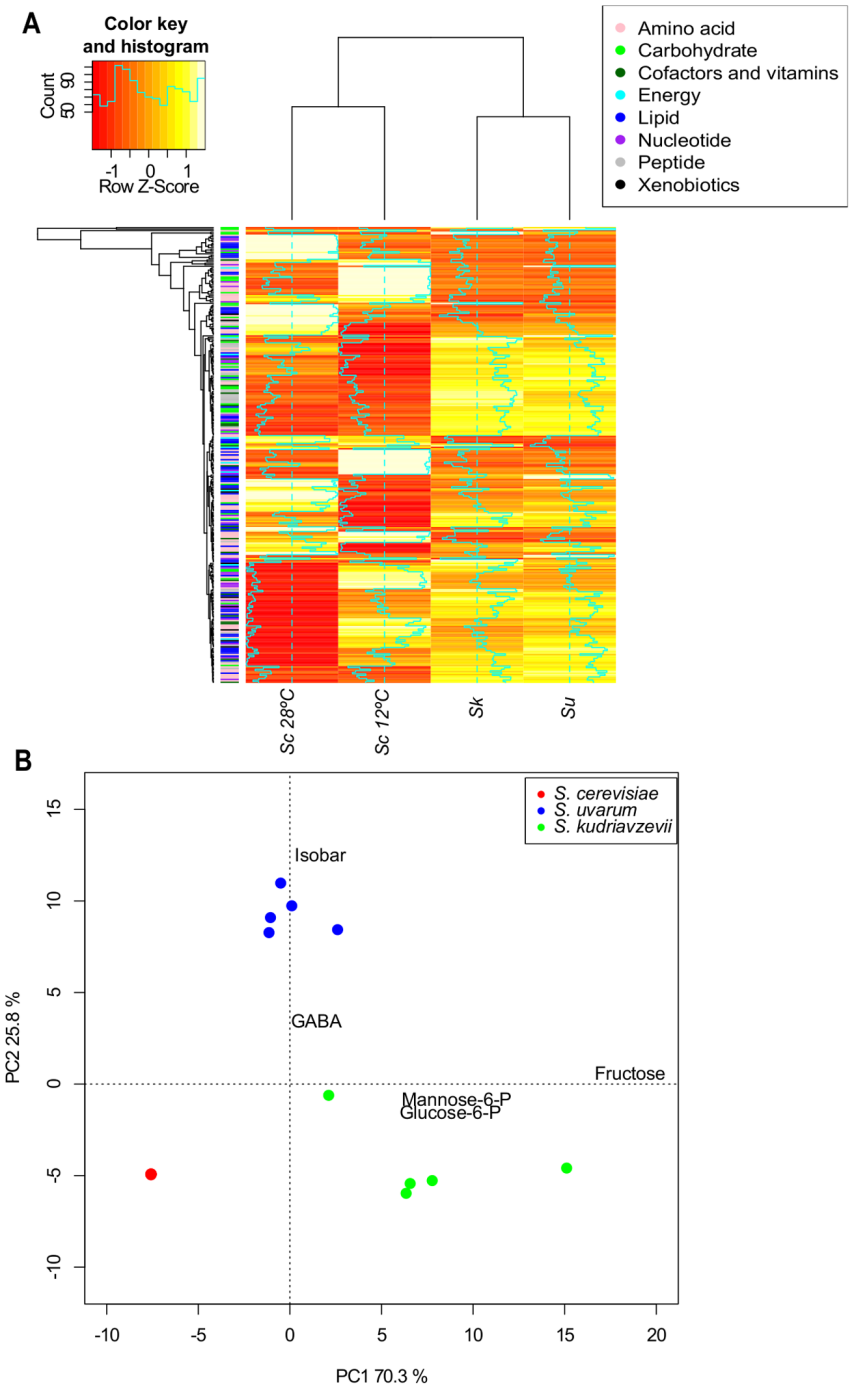
Global biochemical profiles comparison made of the three species. **A**) Hierarchical clustering of all the species is computed after standardizing metabolites to the Z-score. **B**) Biplot of the first two components of the PCA according to the metabolic composition. Isobar  =  isobaric compounds fructose 1,6-diphosphate, glucose 1,6-diphosphate and myo-inositol 1,4 or 1,3 diphosphate.

The differences in metabolite concentration were used to perform a hierarchical clustering (Fig. 1A), grouping to *Sc*−12°C and *Sc*−28°C in a sub-cluster and *Su* and *Sk* in another one. In fact, cryotolerant strains showed a very similar metabolic profile, with increases and decreases in the same metabolic compounds. Although clear differences can be observed in the metabolic profile of *Sc* at both temperatures, differences between this species and the two cryotolerant species were greater. In order to detect the metabolites which better defined the metabolic profiles of these three species, we used a principal component analysis (PCA) (Fig. 1B). The two first components were retained, explaining 96.1% of total variance. The first component (PC1) explained 70.3% of variation and was marked by high positive component loadings for fructose (+0.88), glucose-6-P (+0.31) and mannose-6-P (+0.309). The second component (PC2) explained 25.8% of variation and was marked by high positive component loadings for isobaric compounds fructose 1,6-diphosphate, glucose 1,6-diphosphate and myo-inositol 1,4 or 1,3 diphosphate (+0.938), and for nitrogen compound gamma-aminobutyrate (GABA) (+0.25). No highly negative component loadings were observed for either of both axes. These results indicate that the three species were separated by their differences in carbohydrate metabolism, mainly glucose and fructose metabolism. *Sk* samples were distributed in the lower right quadrant and were characterized by higher concentration of fructose, glucose 6-P and manose 6-P whereas the *Su* samples were situated in the upper quadrant and showed higher concentration of the isobaric compounds (Fig. 1B).

**Figure 2:**
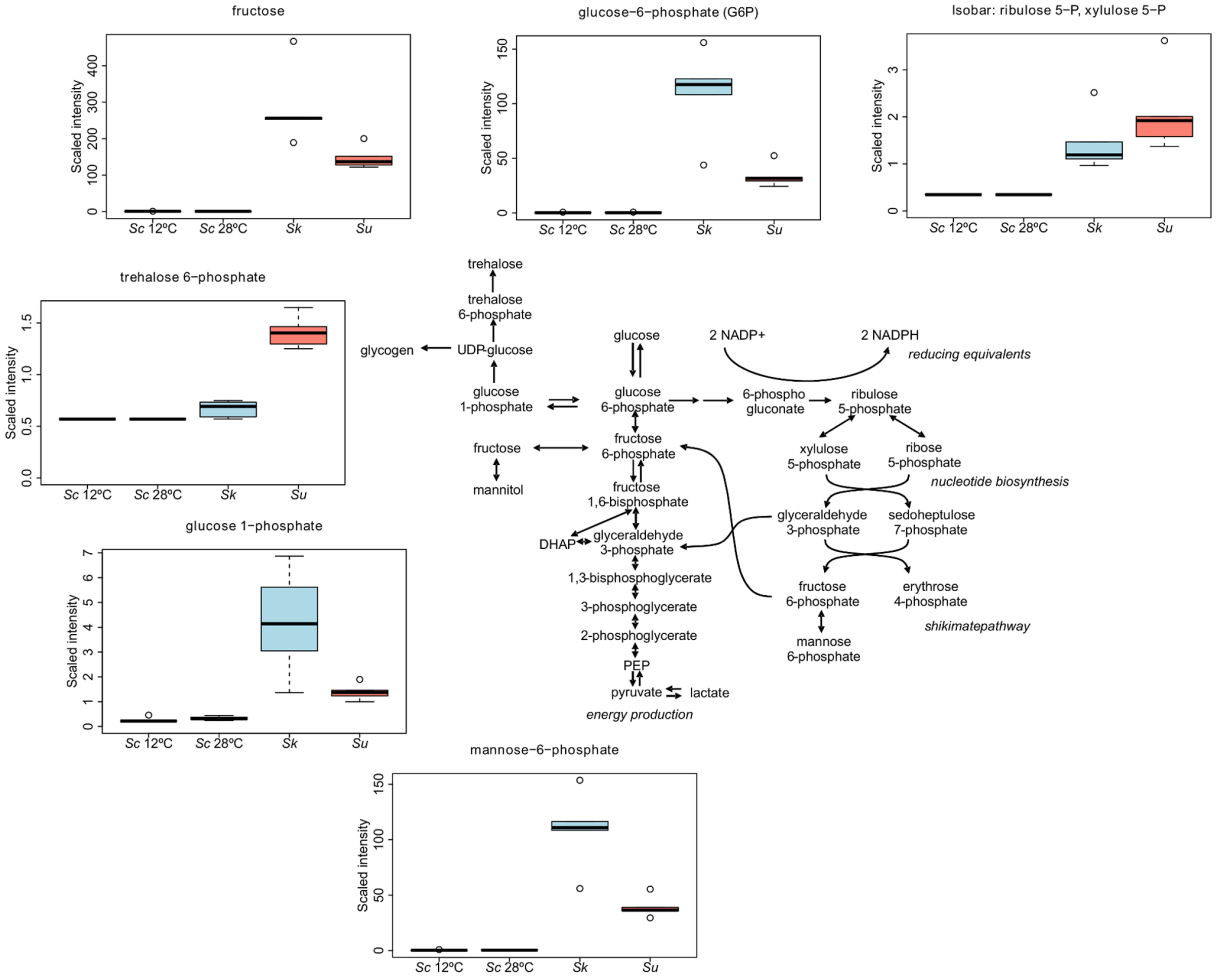
Carbohydrate metabolism: glycolytic pathway, trehalose synthesis, intermediates of the pentose phosphate pathway and protein mannosylation. Differentially produced metabolites within *Su* and *Sk* compared to *Sc*. Box legend: bar inside the box represents the median value, upper bar represents maximum of distribution, lower bar represents minimum of distribution, and the circle represents extreme data points.

The increased levels of these and other compounds of the carbohydrate metabolism are also shown in Fig. 2. Trehalose, but mainly its intermediates glucose-1-phosphate and trehalose-6-phoshate, significantly increased in the cryotolerant species at low temperature. Likewise, intermediates of the pentose phosphate pathway (i.e., isobaric compounds ribulose-5-phosphate and xylulose-5-phosphate), glycolysis (glucose-6-phosphate) and protein mannosylation (mannose-6- phosphate) pathways were all elevated in both *Su* and *Sk* (Fig. 2).

### Comparison of the metabolic profile of the *Sc* growing at 12°C and 28°C

**Figure 3:**
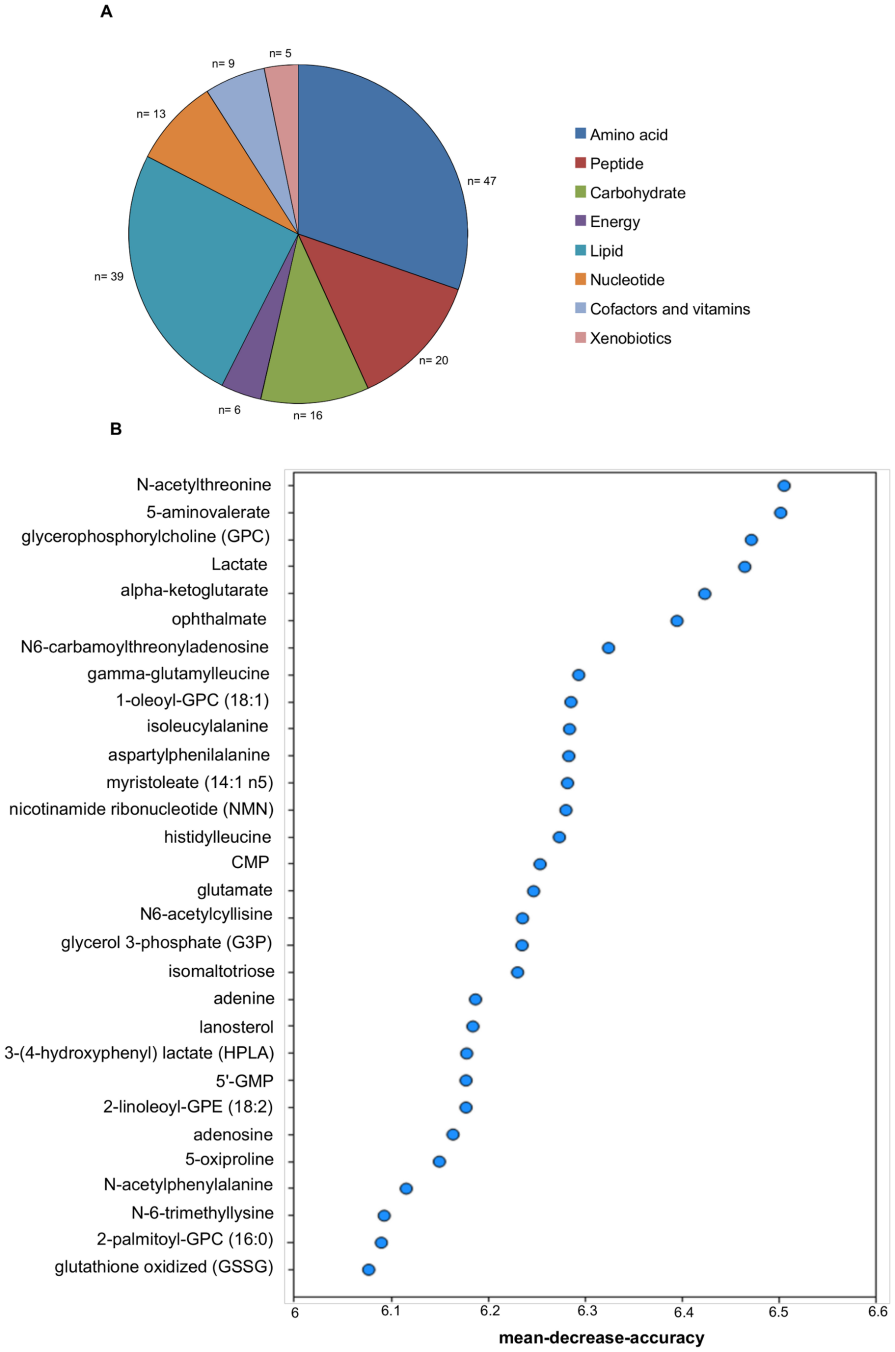
Principal metabolic differences between *Sc* growing at 12°C and at 28°C. A) Class distribution of the different identified metabolites. n  =  number of metabolites in each class. B) The Random Forest statistical analysis of the metabolomic data was used to identify the top 30 biochemicals with the greatest influence in distinguishing different groups. Metabolites are listed on the y-axis in order of importance, which importance decreasing from top to bottom. The mean decrease in accuracy for each metabolite is plotted on the x-axis.

When the metabolic profiles of *Sc* growing at 12°C and 28°C were compared, the statistical t-test showed that more than half the 295 detected biochemicals had significantly changed. These compounds included a wide variety of classes. The majority of differences were observed in the amino acid and lipid classes (Fig. 3A). Another statistical method, known as Random Forest classification, was used to narrow down this large number of changes to a list of biochemicals, which was predicted to have the greatest influence on distinguishing between the *Sc* grown at both temperatures. The Random Forest classification identified a set of metabolites that could be used to separate the two growing conditions with 100% predictive accuracy (Fig. 3B). Among the top 30 metabolites contributing to the classification result, many were connected to membrane lipid metabolism and redox homeostasis.

**Figure 4:**
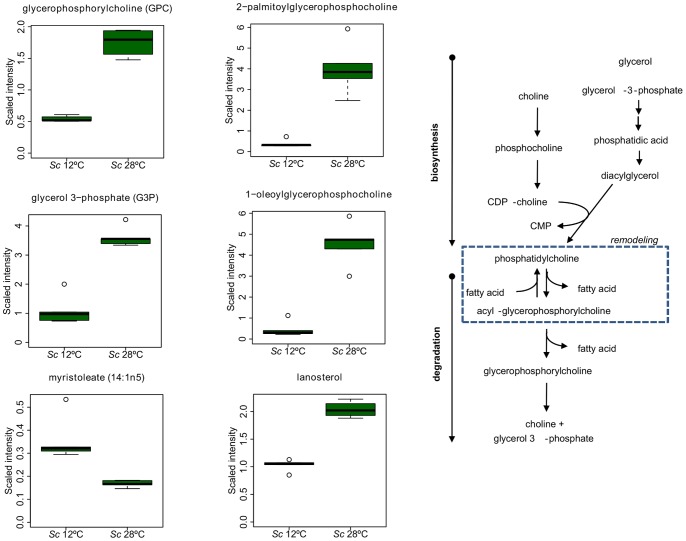
Phosphatidylcholine biosynthesis and degradation in *S. cerevisiae*. Metabolic differences in the *Sc* growing at 12°C and 28°C. Box legend: bar inside the box represents the median value, upper bar represents maximum of distribution, lower bar represents minimum of distribution and the circle represents extreme data points.

Preservation of membrane fluidity is a key cold adaptive response and can be accomplished by increasing the degree of phospholipid acyl-chain unsaturation and by shortening the chain length of these fatty acids. However, only a few unsaturated fatty acids decreased at 28°C (i.e., myristoleate C14∶1 and palmitoleate C16∶1), and several more increased, such as linoleate (C18∶2) and linolenate (C18∶3) (Table S1). We also observed elevated lanosterol levels, intermediates of sterol biosynthesis, in the *Sc* cells grown at 28°C. In addition, the *Sc* grown at optimal temperatures displayed high levels of glycerophosphorylcoline (GPC), 2-palmitoylglycerophosphocholine, glycerol-3-phosphate and 1-oleoyglycerophosphocholine (Fig. 4).

Furthermore, we observed relative elevations in 5-oxoproline, reduced glutathione, gamma-glutamylcysteine and ophthalmate, as well as a relative reduction of oxidized glutathione in the *Sc* cells grown at 28°C, indicating that the cells grown at the higher temperature are better poised to deal with oxidative stress or that the *Sc* growing at 12°C undergo more oxidative stress (Fig. S1).

### Comparison of the metabolic profile of *Sc* and *Su* grown at 12°C

**Figure 5:**
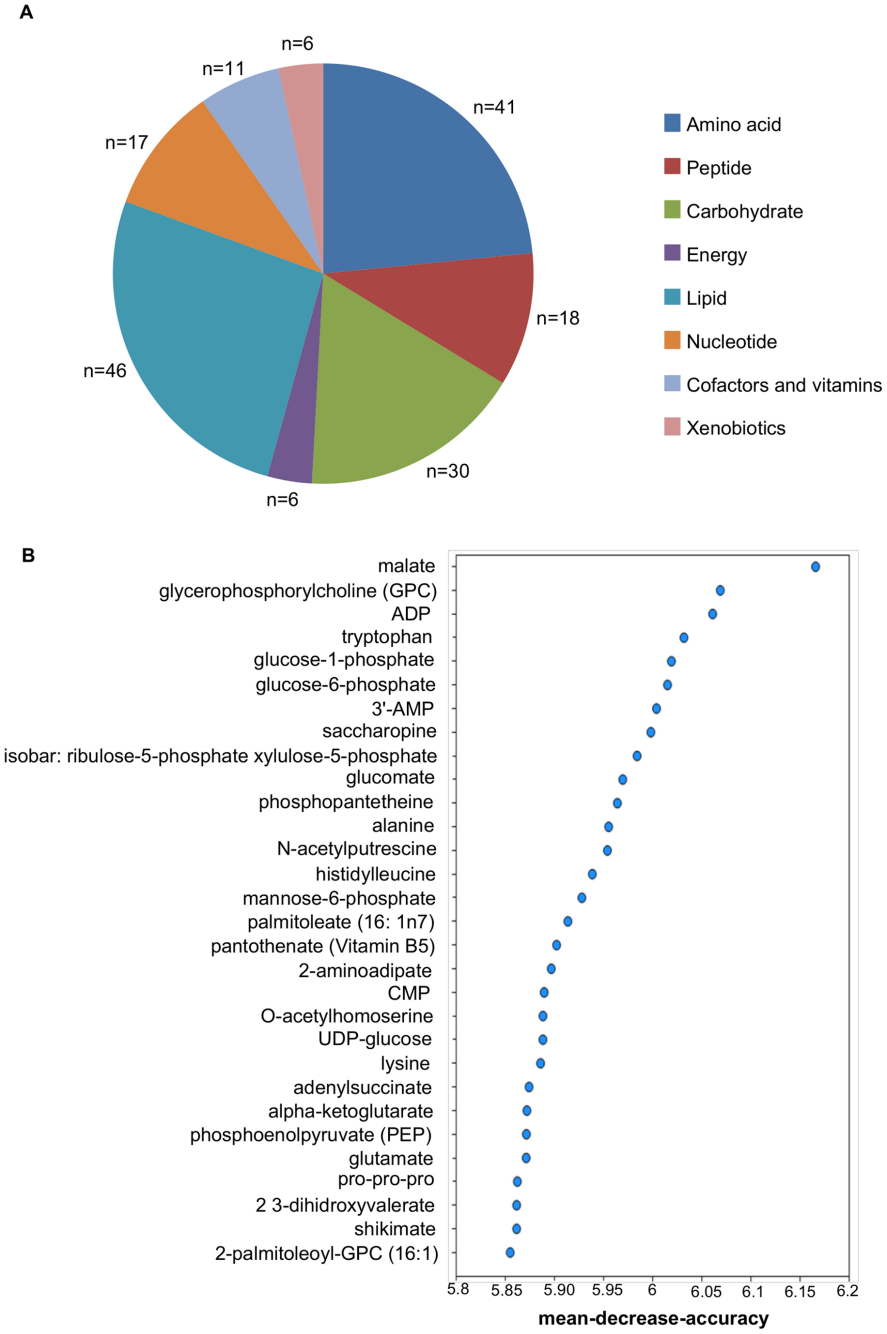
Principal metabolic differences between *Sc* and *Su* at 12°C. A) Class distribution of the different identified metabolites. n  =  number of metabolites in each class. B) The Random Forest statistical analysis of metabolomic data was used to identify the top 30 biochemicals with the greatest influence in distinguishing the different groups. Metabolites are listed on the y-axis in order of importance, with importance decreasing from top to bottom. The mean decrease in accuracy for each metabolite is plotted on the x-axis.

**Figure 6:**
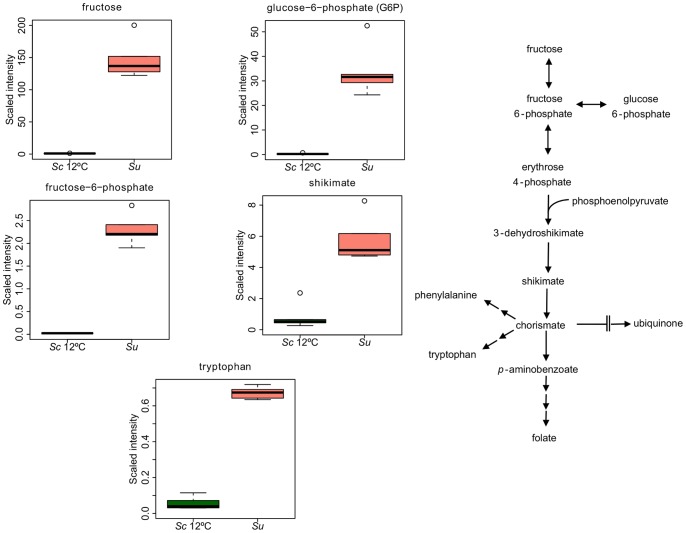
The Shikimate pathway. Differentially produced metabolites within *Su* and *Sc*. Box legend: bar inside the box represents the median value, upper bar represents maximum of distribution, lower bar represents minimum of distribution and the circle represents extreme data points.

In the same way as in the comparison of the metabolic profile of *Sc* at both temperatures, the statistical t-test revealed that more than half the 295 detected biochemicals had significantly changed. In this case, major differences were observed for amino acids, lipids and carbohydrates (Fig. 5A). Moreover, the Random Forest classification identified multiple metabolites connected to the fructose metabolism as discriminating factors to separate *Su* from *Sc* growing at 12°C (Fig. 5B). We also observed that shikimate, its metabolic intermediates, glucose-6-phospate and phosphoenolpyruvate, and a terminal product, tryptophan, were identified by the Random Forest analysis (Fig. 5B), all of them significantly increasing in *Su* (Fig. 6).

In addition, lysine and multiple precursors, including 2-aminoadipate, alpha-ketoglutarate and glutamate, were identified by the Random Forest analysis as compounds that separate these two species (Fig. 5B). Except for alpha-ketoglutarate, the initial substrate of the pathway, lysine and its pathway intermediates, such a 2-aminodipate, saccharopine and homocitrate, were significantly reduced in *Su* (Fig. S2).

### Comparison of the metabolic profile of the *Sc* and *Sk* grown at 12°C

**Figure 7:**
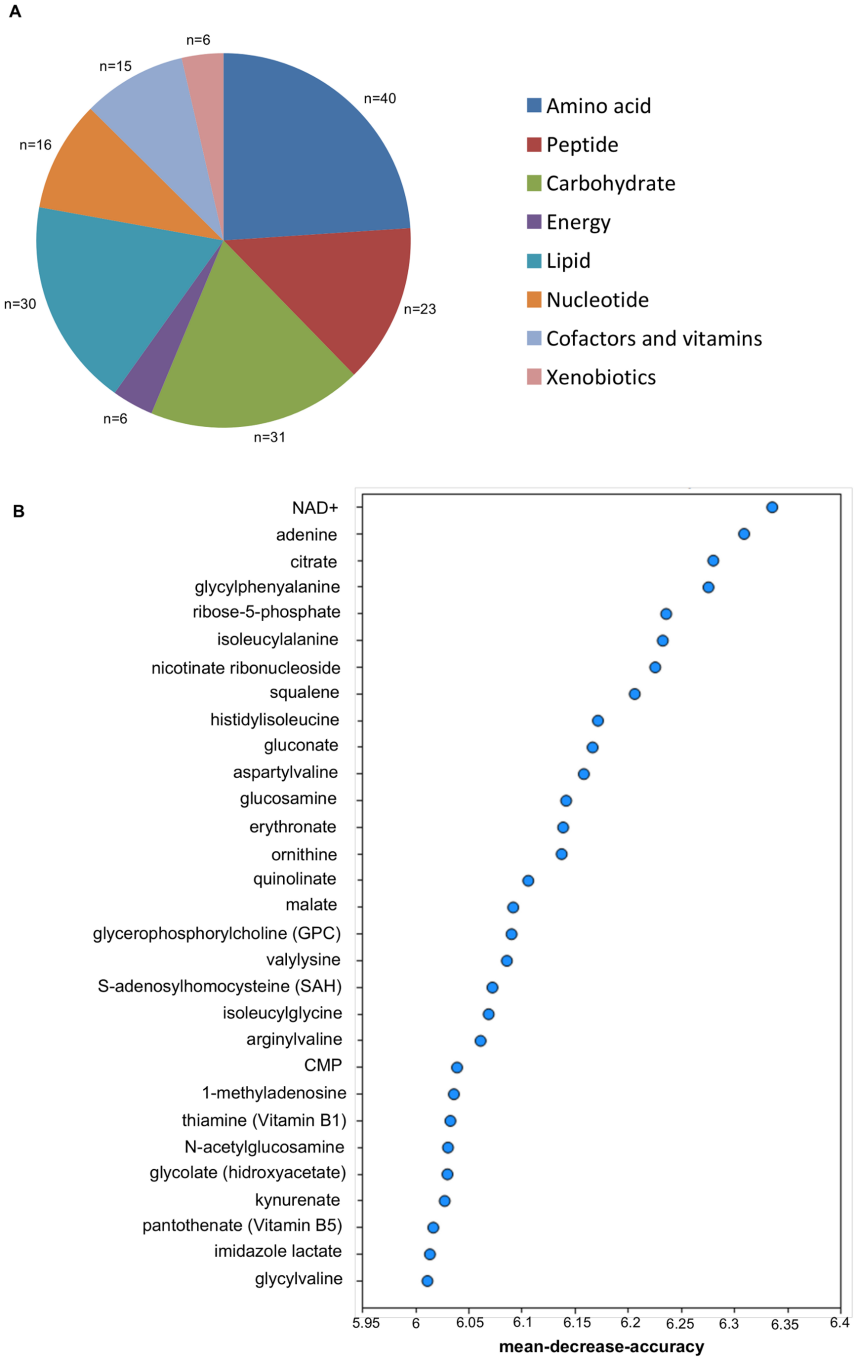
Principal metabolic differences between *S. cerevisiae* and *S. kudriavzevii* growing at 12°C. A) Class distribution of the different identified metabolites. n  =  number of metabolites in each class. B) The Random Forest statistical analysis of metabolomic data was used to identify the top 30 biochemicals with the greatest influence in distinguishing the different groups. Metabolites are listed on the y-axis in order of importance, with importance decreasing from top to bottom. The mean decrease in accuracy for each metabolite is plotted on the x-axis.

In the case of *Sk,* the statistical t-test showed that 179 of the 295 detected biochemicals had significantly changed as compared with *Sc*. Major differences were detected for amino acids, carbohydrates and lipids (Fig. 7A). Moreover, the Random Forest classification identified multiple metabolites connected to the NAD^+^ and chitin biosynthesis as discriminating factors to separate both species (Fig. 7B).

**Figure 8:**
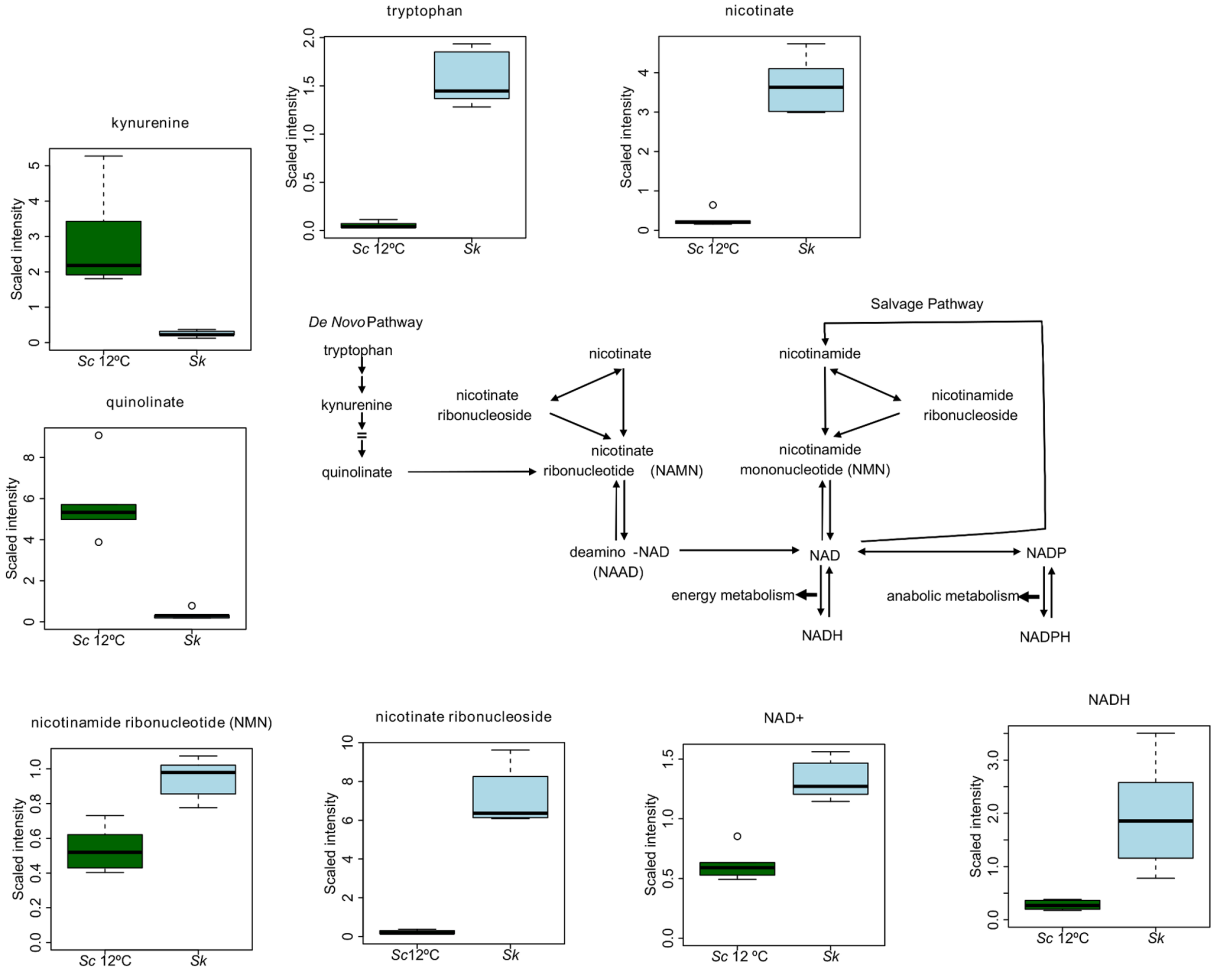
The NAD^+^ biosynthesis pathway. Differentially produced metabolites within *Sk* and *Sc*. Box legend: bar inside the box represents the median value, upper bar represents maximum of distribution, lower bar represents minimum of distribution and the circle represents extreme data points.

NAD^+^ can be synthesized by *de novo* synthesis originating with tryptophan or by the conversion of vitamins nicotinate and nicotinamide. Multiple compounds representing each of these arms of NAD^+^ production showed different concentration in *Sk* (Fig. 8). Elevated tryptophan and the corresponding decreases in kynurenine and quinolinate suggest that the *de novo* synthesis of NAD^+^ has a blockage in the first steps of the route in *Sk*. On the other hand, the increases in nicotinate, nicotinate ribonucleoside and nicotinamide ribonucleotide point to the salvage pathway as responsible for the elevations of NAD^+^ and NADH observed (Fig. 8).

Multiple compounds, including glucosamine, N-acetylglucosamine and erythronate, related to the synthesis of cell wall polysaccharide chitin were identified by the Random Forest classification as separating factors for *S. kudriavzevii* (Fig. 7B and Fig. S3).

## Discussion

Temperature is one of the most important parameters affecting the length and rate of alcoholic fermentation and final wine quality. Many winemakers prefer low-temperature fermentation (10–15°C) for the production of white and “rosé” wines because it improves the taste and aroma characteristics [1], [2]. Although *S. cerevisiae* is always predominant in wine fermentations, a drop in temperature affects its competitiveness. It is obvious that the direct effect of lowering temperature is to slow down the metabolic activity of the yeast cell, which accounts for reduced growth and longer fermentation process at 12°C. Thus, we characterized the metabolome of a commercial wine strain *Sc* growing at 12°C and 28°C. A metabolome analysis is a powerful tool to understand metabolic changes in response to different environmental conditions, such as cold temperatures. In past years, some attempts have been made to elucidate the cold response of the same wine yeast *Sc* using a variety of high-throughput methodologies, such as transcriptomics [21] or proteomics [30]. These metabolic changes could be due to temperature-mediated transcriptional and/or to post-transcriptional effects on yeast. However, multiple genes may be involved in the synthesis and degradation of a single metabolite. This makes the metabolome an appropriate level for studying phenotypic responses.

Notwithstanding, the fact that *S. cerevisiae* is the predominant species responsible for alcoholic fermentation, other species of the genus *Saccharomyces*, such as *S. bayanus* var. *uvarum* [6] or the hybrid strains of *S. cerevisiae* x *S. kudriavzevii* [7], have shown to be better adapted at low temperatures during winemaking. Thus, these two species are often referred to as being cryotolerant [9], [3] and are good models to study adaptation at low temperature. To this end, we also characterized the metabolome of *Su* and *Sk* growing at 12°C, which we compared with the metabolome of *Sc* (not well-adapted at low temperature) at 12°C. This comparison revealed that the inter-specific differences were even greater than the temperature-dependent metabolic changes. As we did not analyze the metabolic profiling of *Su* and *Sk* at 28°C, we cannot ascertain whether the changes observed in the metabolism of these species were due to low temperature. In a future study, the metabolic adaptation of these cryotolerant species at low temperature should be dealt with. The main differences between the metabolic profiling of *Sc* growing at 12°C and 28°C were observed in amino acid and lipid classes. These differences in the amino acid metabolism may be connected with the different consumption rate of organic nitrogen at both temperatures (Table 1). Several studies have reported the importance of lipid composition in the yeast adaptive response at low temperature [1], [2], [31], [32]. Furthermore, Tai *et al.* [18] observed that the only clear group of genes that were commonly regulated in a low-temperature chemostat and batch-culture studies was involved in lipid metabolism. Incubation at low temperature increases the molecular order of membrane lipids by rigidification [33]. Yeasts are known to have developed several strategies to maintain appropriate membrane fluidity. The most commonly studied involves increased unsaturation and reduced average chain length (ChL) of fatty acids (FA) [1], [2]. Our data reveal that the cells growing at low temperature increase the unsaturated fatty acids (UFA) of C14 and C16 (myristoleate and palmitoleate), but decrease other unsaturated fatty acids (linoleate C18∶2 and linolenate C18∶3). The result is a global reduction in the chain length of FA as a main strategy to enhance membrane fluidity. The increase in the medium chain FA as a caprylate (C8∶0) in the cells cultured at 12°C also supports the predominance of this strategy.

Our results also show lower levels of the metabolites connected with phosphatidilcholine (PC), such as glycerophosphorylcoline (GPC), 2-palmitoylglycerophosphocholine and 1-oleoyglycerophosphocholine, in the *Sc* grown at 12°C. Redon *et al*. [32] and Tronchoni *et al*. [34] also reported a lowered ratio of phosphatidilcholine (PC)/phosphatidylethanolamine (PE) when this species was cultured at low temperature. Regarding the sterol pathway, the strategy for counteracting the membrane rigidity in the cells grown at 12°C was to increase in ergosterol, but to decrease in their intermediates squalene and lanosterol.

Another set of metabolites that distinguished the *Sc* grown at 12°C and 28°C were the metabolites belonging to the glutathione/glutaredoxin system, biochemicals that help cells detoxify reactive oxygen species (ROS). Cross-talk between signal transduction in response to the temperature downshift and oxidative stress has been previously reported by Zhang *et al*. [35], who observed that the mRNA levels of *SOD1*, *CTT1* and *GSH1* significantly rose by a temperature downshift from 30°C to 10°C. Moreover, and consistent with this idea, Schade *et al*. [16] showed that the transcriptional profile of the glutathione/glutaredoxin system genes suggests activation during the LCR (Late Cold Response). In a proteomic study of the response of the same wine yeast at low temperature, Salvadó *et al*. [30] also detected a higher concentration of Yhb1 (Yeast hemoglobin-like protein), with functions in oxidative stress response.

The global metabolic comparison made of the three species revealed that the main differences between the two cryophilic species and *S. cerevisiae* were in the carbohydrate metabolism. The *Sk* and *Su* strains had significantly higher levels of glucose and fructose, and most of the intermediates were of the higher part of glycolysis (C6 sugars), the pentose phosphate pathway and the trehalose metabolism. The easiest explanation is that these strains present a higher sugar uptake at low temperature. Yet when considering the residual sugars in the supernatant of steady-state cultures (Table 1), *Sk* and *Su* had consumed less sugars than *Sc* at both temperatures. It is well-known that *Sc* is the species with the greatest fermentative competitiveness. This fitness advantage has been related with quicker sugar uptake and speedier flux by the glycolysis pathway than its competitors [36], thus enabling better ethanol yields, which allow niche construction via ethanol production [13]. The comparison made of sugar consumption and ethanol production rates clearly confirmed the enhanced fermentative performance of *Sc* in comparison to *Su* and *Sk* (Table 1). Thus, the higher concentration of glucose, fructose and the intermediates of other biochemical pathways in the *Sk* and *Su* strains may be related with a slower glycolytic flux, mainly at the level of conversion of hexoses into trioses (higher concentration in *Sc*).

Despite the main differences among the three species lying in the carbohydrate metabolism, other important differences have been be detected. Shikimate, its metabolic intermediates, glucose-6-phosphate and phosphoenolpyruvate, which are shared with the glycolysis pathway, and a terminal product, tryptophan, were identified by the Random Forest analysis as factors separating *Su* from *Sc*. Shikimate is an important precursor for aromatic amino acids tyrosine, phenylalanine and tryptophan. It is well-documented that tryptophan uptake is a limiting factor for yeast cell growth at low temperature [37]. These authors postulated that the increased rigidity of the plasma membrane at low temperature especially impairs tryptophan transport, and they also proved that the overexpression of high-affinity tryptophan transporter Tat2p improves cell growth at low temperature [37]. Thus, the increased tryptophan biosynthesis in *Su* can counteract the transport problem and improve its growth at low temperature.

Another amino acid biosynthesis route also showed differences in its intermediates and the final product separating the *Su* and *Sc* strains. Apart from alpha-ketoglutarate, the initial substrate of the pathway, lysine and its pathway intermediates (e.g., 2-aminoadipate, saccharopine, and homocitrate) significantly reduced in *S. bayanus* var. *uvarum*. Homocitrate synthase (HCS) catalyzes the conversion of alpha-ketoglutarate into homocitrate, and represents the rate-limiting step of lysine synthesis. The observed pattern of changes strongly suggests that HCS, a highly regulated enzyme, activity is inhibited in *S. bayanus* var. *uvarum.* Yet no obvious relationship to its ability to grow at 12°C has been identified in the scientific literature.

Regarding *Sk*, it is characterized by increased NAD^+^ synthesis and cell wall synthesis. NAD^+^ is a primary cofactor for the reductive reactions of energy metabolism and can serve as a marker of cellular energy status. Additionally, it is a co-substrate for certain NAD^+^-dependent histone deacetylases of the sirtuin family, which play a role in gene silencing and in regulating cellular metabolism [38]. The relative elevation of NAD^+^ and NADH suggest that *Sk* has considerable nucleotide cofactor capacity to catalyze the reactions related to energy metabolism, as well as those reactions controlled by NAD^+^-dependent sirtuins. Furthermore, *Sk* has elevated several compounds relating to the synthesis of the cell wall polysaccharide chitin. It is known that many cold shock proteins in *S. cerevisiae* are serine/threonine-rich mannoproteins localized in the cell wall [17], [39], [40]. Thus, cell wall composition appears to be important during cold adaptation.

In summary, with this global metabolic profiling study, we have detected the main metabolites involved in low temperature response in three different yeast species from the genus *Saccharomyces.* We have confirmed the importance of lipid metabolism in the cold adaptive response in *S. cerevisiae*, especially the lesser amount of phosphatidylcholine and its derivatives. We have also observed that two cryotolerant species, *S. bayanus* var. *uvarum* and *S. kudriavzevii*, differ from *S. cerevisiae* in terms of their capacity to use fructose at 12°C. Nevertheless, these two species have developed different strategies for cold resistance. *S. bayanus* var. *uvarum* showed great shikimate pathway activity, while *S. kudriavzevii* presented increased NAD^+^ synthesis. The differences in these metabolites of cold tolerance and the winemaking process suggest that it is possible to segregate the metabolism of *S. cerevisiae* from that of *S. uvarum* and *S. kudriavzevi*, and that it may provide a basis for understanding why *S. cerevisiae* performs better at 28°C, while its *Saccharomyces* counterparts do better at 12°C. This study advances our understanding of the cold response within the *Saccharomyces* genus at the metabolic level and can be used as a basis for future biotechnological applications for low-temperature wine fermentations.

## Supporting Information

Figure S1 **The homeostasis redox in** ***S. cerevisiae*** **.** Metabolic differences in the *Sc* growing at 12°C and 28°C. Box legend: bar inside the box represents the median value, upper bar represents maximum of distribution, lower bar represents minimum of distribution and the circle represents extreme data points.
(TIFF)

Figure S2 **Lysine synthesis.** Differentially produced metabolites within *Su* and *Sc*. Box legend: bar inside the box represents the median value, upper bar represents maximum of distribution, lower bar represents minimum of distribution and the circle represents extreme data points.
(TIFF)

Figure S3 **Cell wall synthesis.** Differentially produced metabolites within *Sk* and *Sc*. Box legend: bar inside the box represents the median value, upper bar represents maximum of distribution, lower bar represents minimum of distribution and the circle represents extreme data points.
(TIFF)

Table S1 **Metabolic comparison between** ***Sc*** **growing at 28°C,** ***Su*** **and** ***Sk*** **and** ***Sc*** **growing at 12°C (control condition).** Green indicates difference (p≤0.05) between the groups shown, indicating a ratio <1. Light green indicates approaching significance (0.05≤p≤0.1) between the groups shown, indicating a ratio <1. Red indicates difference (p≤0.05) between the groups shown, indicating a ratio >1. Pink indicates approaching significance (0.05≤p≤0.1) between the groups shown, indicating a ratio >1. Non-coloured cell means values which are not significantly different for the comparison.
(DOC)
