# Supplementary figures and images for: Metabolomic Comparison of Saccharomyces cerevisiae and the Cryotolerant Species S. bayanus var. uvarum and S. kudriavzevii during Wine Fermentation at Low Temperature
Source: PLoS One. 2013 Mar 20;8(3):e60135. doi: 10.1371/journal.pone.0060135 (PMC3603904; doi:10.1371/journal.pone.0060135)

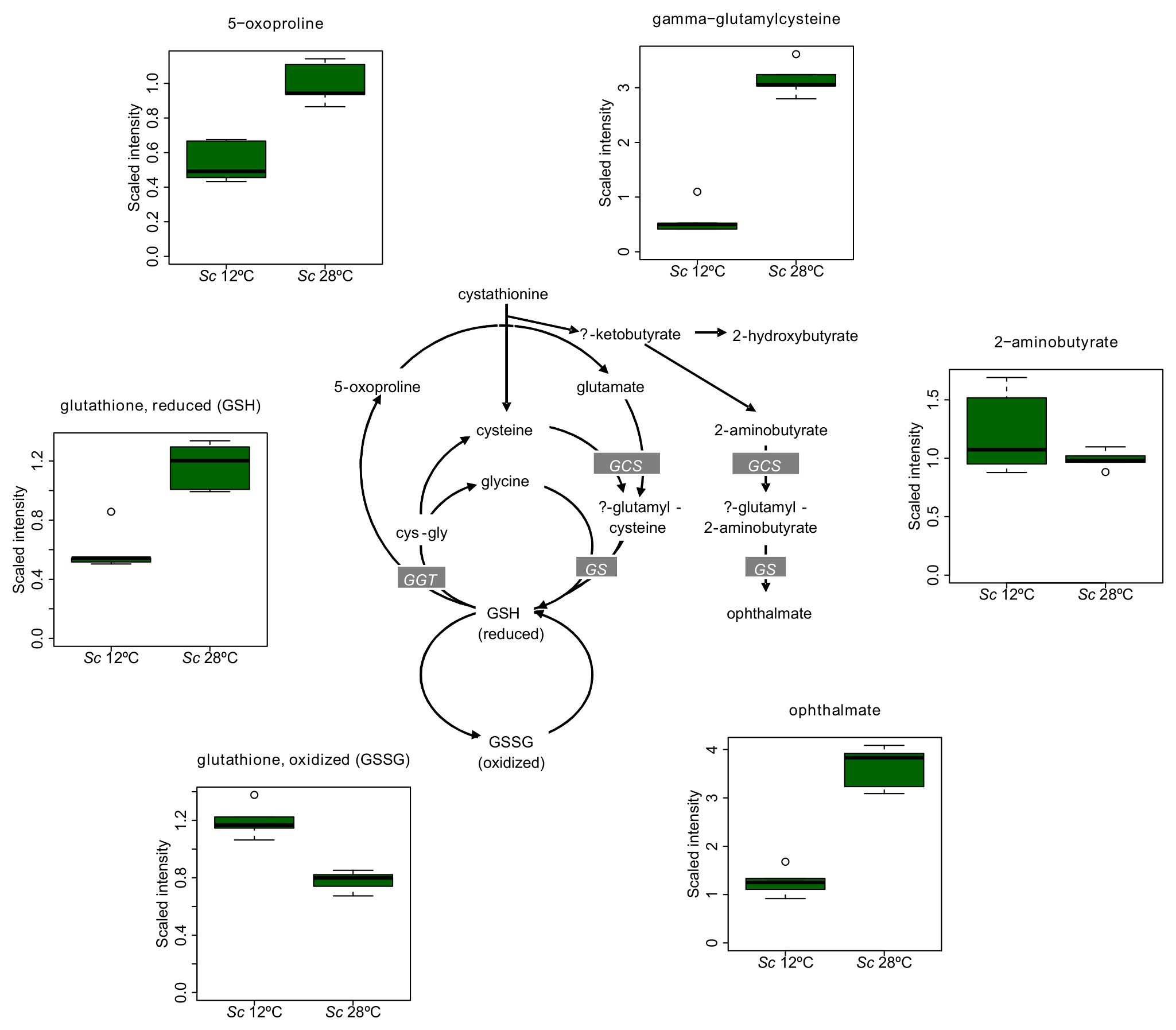

Supplement: Figure S1 — The homeostasis redox in S. cerevisiae . Metabolic differences in the Sc growing at 12°C and 28°C. Box legend: bar inside the box represents the median value, upper bar represents maximum of distribution, lower bar represents minimum of distribution and the circle represents extreme data points. (TIFF) [file pone.0060135.s001.tiff]

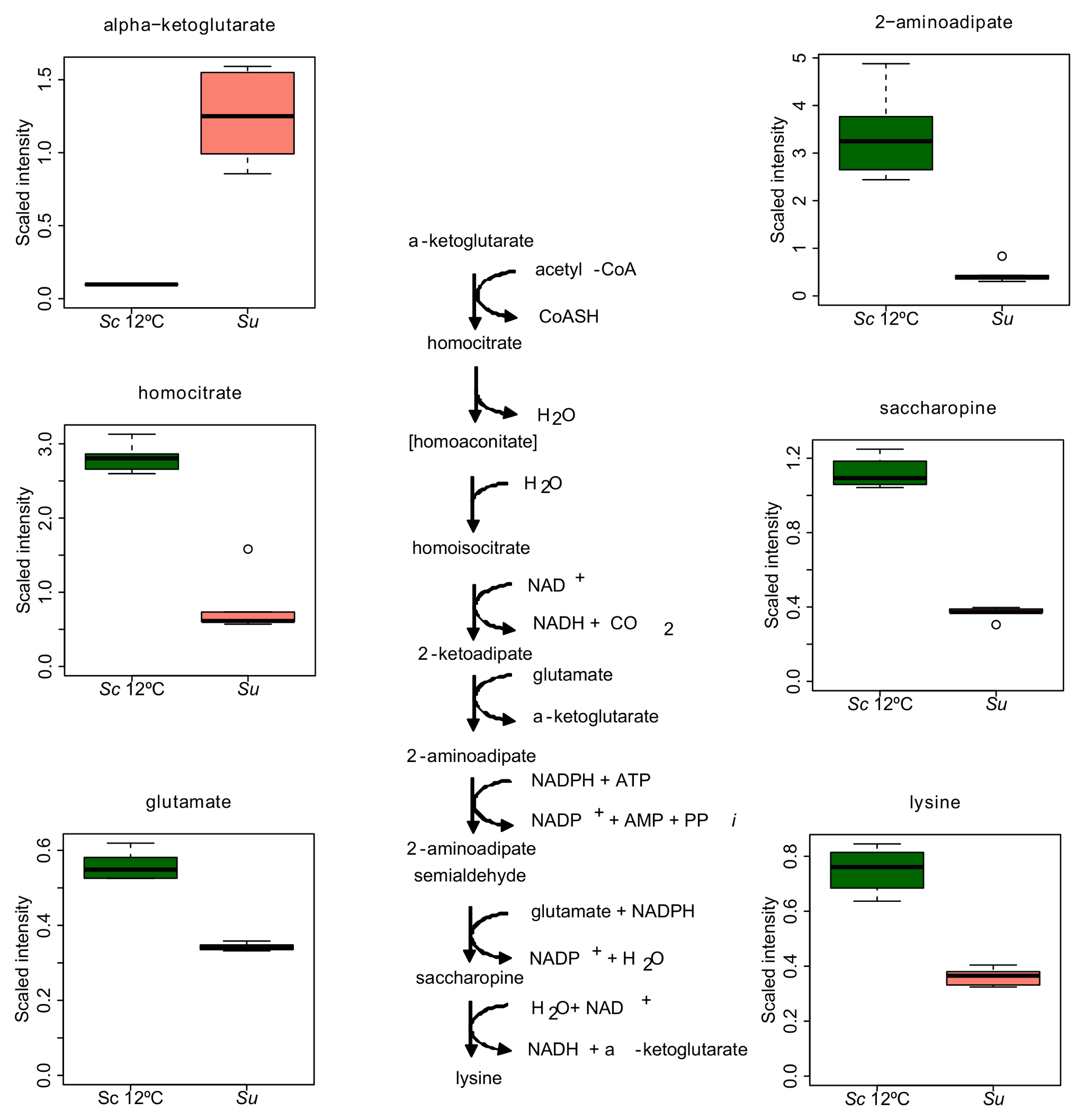

Supplement: Figure S2 — Lysine synthesis. Differentially produced metabolites within Su and Sc. Box legend: bar inside the box represents the median value, upper bar represents maximum of distribution, lower bar represents minimum of distribution and the circle represents extreme data points. (TIFF) [file pone.0060135.s002.tiff]

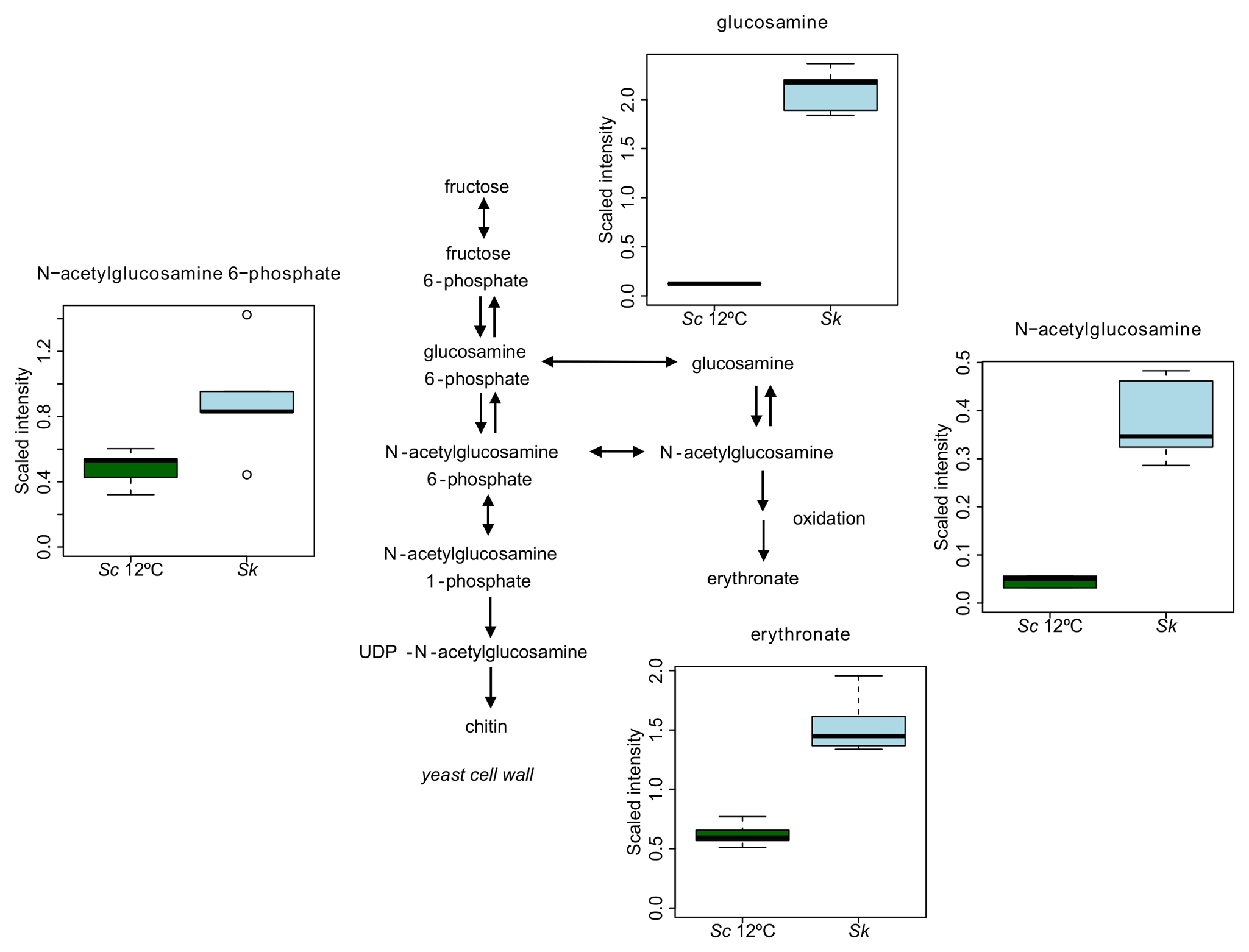

Supplement: Figure S3 — Cell wall synthesis. Differentially produced metabolites within Sk and Sc. Box legend: bar inside the box represents the median value, upper bar represents maximum of distribution, lower bar represents minimum of distribution and the circle represents extreme data points. (TIFF) [file pone.0060135.s003.tiff]
