# Supplementary material for: Metabolomic Comparison of Saccharomyces cerevisiae and the Cryotolerant Species S. bayanus var. uvarum and S. kudriavzevii during Wine Fermentation at Low Temperature
Source: PLoS One. 2013 Mar 20;8(3):e60135. doi: 10.1371/journal.pone.0060135 (PMC3603904; doi:10.1371/journal.pone.0060135)
Supplement: Table S1 — Metabolic comparison between Sc growing at 28°C, Su and Sk and Sc growing at 12°C (control condition). Green indicates difference (p≤0.05) between the groups shown, indicating a ratio <1. Light green indicates approaching significance (0.05≤p≤0.1) between the groups shown, indicating a ratio <1. Red indicates difference (p≤0.05) between the groups shown, indicating a ratio >1. Pink indicates approaching significance (0.05≤p≤0.1) between the groups shown, indicating a ratio >1. Non-coloured cell means values which are not significantly different for the comparison. (DOC) [file pone.0060135.s004.doc]

**Table S1.** Metabolic comparison between *Sc* growing at 28 ºC, *Su* and *Sk* and *Sc* growing at 12 ºC (control condition). Green indicates difference (p≤0.05) between the groups shown, indicating a ratio < 1. Light green indicates approaching significance (0.05≤p≤0.1) between the groups shown, indicating a ratio < 1. Red indicates difference (p≤0.05) between the groups shown, indicating a ratio > 1. Pink indicates approaching significance (0.05≤p≤0.1) between the groups shown, indicating a ratio > 1. Non-coloured cell means values which are not significantly different for the comparison.

|  |  |  |  | |  | | |  |  |  |
| --- | --- | --- | --- | --- | --- | --- | --- | --- | --- | --- |
|  |  |  |  | | **Fold of Change** | | |  |  |  |
|  |  |  |  | | **Sc QA23-28C** | | | **Su-12C** | **Sk-12C** | **Sk-12C** |
|  |  |  |  | |  | | |  |  |  |
| **Super Pathway** | **Sub Pathway** | **Biochemical Name** | **KEGG** | | **Sc QA23-12C** | | | **Sc QA23-12C** | **Sc QA23-12C** | **Su-12C** |
| Amino acid | Glycine, serine and threonine metabolism | Glycine | C00037 | | **1,35** | | | **0,62** | 0,79 | 1,27 |
|  |  | N-acetylglycine |  | | 1,04 | | | 0,82 | **3,59** | **4,38** |
|  |  | beta-hydroxypyruvate | [C00168](http://www.genome.jp/dbget-bin/www_bget?cpd+C00168) | | 1 | | | 1 | **1,54** | **1,54** |
|  |  | Serine | C00065 | | **1,88** | | | 1,31 | 1,18 | 0,9 |
|  |  | N-acetylserine |  | | 1,14 | | | **0,35** | 0,62 | **1,77** |
|  |  | Homoserine | C00263,C02926 | | **2,19** | | | 0,71 | **2,39** | **3,37** |
|  |  | O-acetylhomoserine | C01077 | | **1,6** | | | **0,3** | **0,08** | **0,27** |
|  |  | Threonine | C00188 | | **2,41** | | | **0,69** | **1,48** | **2,13** |
|  |  | N-acetylthreonine | C01118 | | **0,3** | | | **0,17** | 0,75 | **4,47** |
|  |  | allo-threonine | C05519 | | **3,6** | | | **2,11** | **1,75** | 0,83 |
|  | Alanine and aspartate metabolism | Aspartate | C00049 | | **5,52** | | | **1,83** | **2,5** | **1,37** |
|  |  | Asparagine | C00152 | | **2,81** | | | 0,93 | 1,18 | 1,27 |
|  |  | Alanine | C00041 | | **1,39** | | | **0,56** | 0,99 | **1,77** |
|  |  | N-carbamoylaspartate | C00438 | | 1 | | | 1 | 1 | 1 |
|  | Glutamate metabolism | Glutamate | C00025 | | **2,72** | | | **0,61** | 1,05 | **1,72** |
|  |  | glutamate, gamma-methyl ester |  | | **1,72** | | | **0,26** | **0,25** | 0,95 |
|  |  | glutamine | C00064 | | 0,73 | | | 1,11 | 0,94 | **0,85** |
|  |  | gamma-aminobutyrate (GABA) | C00334 | | **2,61** | | | **93,59** | **20,27** | **0,22** |
|  |  | N-acetylglutamate | C00624 | | **4,57** | | | 1,38 | **1,72** | 1,24 |
|  | Histidine metabolism | histidine | C00135 | | **0,29** | | | **0,27** | **0,44** | **1,63** |
|  |  | N-acetylhistidine | C02997 | | 1 | | | 1 | 1 | 1 |
|  |  | imidazole lactate | C05568 | | **0,09** | | | **0,16** | **0,05** | **0,33** |
|  | Lysine metabolism | glutarate (pentanedioate) | C00489 | | **0,24** | | | **0,56** | **0,37** | 0,66 |
|  |  | lysine | C00047 | | **1,52** | | | **0,48** | 1,17 | **2,43** |
|  |  | N6-carboxyethyllysine |  | | **2,52** | | | 0,63 | 0,63 | 1 |
|  |  | 2-aminoadipate | C00956 | | **0,43** | | | **0,14** | **0,36** | **2,66** |
|  |  | pipecolate | C00408 | | 0,97 | | | **0,41** | **0,22** | **0,54** |
|  |  | N-6-trimethyllysine | C03793 | | **1,81** | | | 0,99 | 0,8 | 0,81 |
|  |  | saccharopine | C00449 | | **1,29** | | | **0,33** | **0,48** | 1,47 |
|  |  | N2-acetyllysine | C12989 | | **1,4** | | | **0,45** | 0,91 | **2** |
|  |  | N6-acetyllysine | C02727 | | **3,46** | | | 1,2 | **1,43** | 1,2 |
|  |  | 2-aminopentanoate |  | | 1,3 | | | **0,32** | **0,48** | 1,51 |
|  | Phenylalanine & tyrosine metabolism | phenyllactate (PLA) | C05607 | | **2,21** | | | **3,94** | 0,7 | **0,18** |
|  |  | phenylalanine | C00079 | | **2,03** | | | 1,24 | 1,23 | 0,99 |
|  |  | tyrosine | C00082 | | **2,43** | | | 0,79 | 0,85 | 1,08 |
|  |  | 3-(4-hydroxyphenyl)lactate | C03672 | | **3,81** | | | **1,92** | 0,89 | **0,46** |
|  |  | 4-hydroxyphenylpyruvate | C01179 | | 1,2 | | | 0,95 | 0,92 | 0,97 |
|  |  | N-acetylphenylalanine | C03519 | | **3,64** | | | 1,48 | 0,69 | **0,46** |
|  |  | N-acetyltyrosine |  | | **2,23** | | | **0,34** | **0,34** | 1 |
|  | Tryptophan metabolism | xanthurenate | C02470 | | **2,49** | | | 0,52 | 0,52 | 1 |
|  |  | kynurenate | C01717 | | **1,45** | | | **0,04** | **0,02** | **0,57** |
|  |  | kynurenine | C00328 | | **1,48** | | | **0,26** | **0,09** | **0,33** |
|  |  | tryptophan | C00078 | | **7,16** | | | **11,74** | **27,5** | **2,34** |
|  |  | anthranilate | C00108 | | **0,65** | | | **0,65** | **0,65** | 1 |
|  |  | 3-hydroxykynurenine | C02794 | | **1,65** | | | **0,15** | 1,45 | **10,02** |
|  |  | 3-hydroxyanthranilate | C00632 | | **0,62** | | | 1,24 | 1,42 | 1,15 |
|  | Valine, leucine and isoleucine metabolism | alpha-hydroxyisocaproate | C03264 | | 1,53 | | | 1,04 | 1,32 | 1,27 |
|  |  | isoleucine | C00407 | | **1,52** | | | 1,06 | 1,26 | 1,19 |
|  |  | leucine | C00123 | | **1,88** | | | 1,14 | 1,09 | 0,95 |
|  |  | N-acetylleucine | C02710 | | 1,4 | | | 1,54 | 1,01 | 0,66 |
|  |  | N-acetylisoleucine |  | | 0,77 | | | 0,91 | 1,3 | 1,44 |
|  |  | valine | C00183 | | **1,54** | | | 1,16 | 1,23 | 1,06 |
|  |  | 4-methyl-2-oxopentanoate | C00233 | | 1,46 | | | 0,56 | 1,14 | 2,03 |
|  |  | alpha-hydroxyisovalerate |  | | **1,6** | | | **2,34** | 1,37 | **0,59** |
|  |  | citramalate | C02612,C00815 | | 1,34 | | | **0,12** | **0,25** | 2,18 |
|  | Cysteine, methionine, SAM, taurine metabolism | cysteine | C00097 | | 1,14 | | | **0,35** | **2,14** | **6,12** |
|  |  | cystathionine | C02291 | | 0,47 | | | **0,13** | 1,21 | **9,19** |
|  |  | S-adenosylhomocysteine (SAH) | C00021 | | 0,91 | | | 0,87 | **6,7** | **7,72** |
|  |  | methionine | C00073 | | **1,77** | | | 1,31 | 1,12 | 0,86 |
|  |  | N-acetylmethionine | C02712 | | 0,74 | | | **0,4** | 0,71 | **1,77** |
|  |  | homocysteine | C00155 | | **1,5** | | | 0,72 | **2,84** | **3,93** |
|  | Urea cycle; arginine-, proline-, metabolism | dimethylarginine (SDMA + ADMA) | C03626 | | 0,94 | | | 0,99 | **2,27** | **2,3** |
|  |  | arginine | C00062 | | **0,44** | | | **0,36** | **2,44** | **6,81** |
|  |  | N-acetylarginine | C02562 | | **0,66** | | | **0,55** | **2,25** | **4,12** |
|  |  | ornithine | C00077 | | **1,53** | | | **0,11** | **0,27** | **2,37** |
|  |  | urea | C00086 | | 0,46 | | | **0,33** | **0,21** | **0,64** |
|  |  | proline | C00148 | | 1,17 | | | 1,16 | **2,32** | **1,99** |
|  |  | 5-aminovalerate | C00431 | | **0,08** | | | **0,28** | **0,18** | **0,64** |
|  |  | citrulline | C00327 | | **15,84** | | | **5,62** | 1,06 | **0,19** |
|  |  | N-acetylornithine | C00437 | | **1,73** | | | 1 | 1 | 1 |
|  |  | argininosuccinate | C03406 | | 0,69 | | | **0,41** | **0,46** | 1,13 |
|  | Butanoate metabolism | 2-aminobutyrate | C02261 | | 0,81 | | | **0,21** | **0,26** | 1,2 |
|  | Polyamine metabolism | 5-methylthioadenosine (MTA) | C00170 | | **4,48** | | | 1,02 | **4,32** | **4,24** |
|  |  | putrescine | C00134 | | **14,54** | | | 0,64 | **0,27** | **0,41** |
|  |  | N-acetylputrescine | C02714 | | **0,58** | | | **0,41** | **0,44** | 1,07 |
|  |  | agmatine | C00179 | | **5,99** | | | 0,62 | 0,56 | 0,9 |
|  |  | spermidine | C00315 | | **2,95** | | | 0,74 | 1,53 | **2,07** |
|  | Guanidino and acetamido metabolism | 4-guanidinobutanoate | C01035 | | 0,82 | | | **0,46** | 0,96 | **2,09** |
|  | Glutathione metabolism | glutathione, reduced (GSH) | C00051 | | **1,97** | | | 0,88 | **1,44** | **1,64** |
|  |  | 5-oxoproline | C01879 | | **1,84** | | | **1,58** | **2,91** | **1,84** |
|  |  | glutathione, oxidized (GSSG) | C00127 | | **0,65** | | | **0,66** | **0,11** | **0,17** |
|  |  | ophthalmate |  | | **2,89** | | | **0,76** | **0,65** | **0,86** |
| Peptide | Dipeptide | glycylvaline |  | | 0,7 | | | 0,75 | **8,3** | **11,08** |
|  |  | glycylglycine | C02037 | | **1,52** | | | **0,34** | 0,87 | **2,6** |
|  |  | glycylproline |  | | **1,51** | | | 0,97 | **1,82** | **1,87** |
|  |  | glycylisoleucine |  | | 1,34 | | | **3,3** | **23,45** | **7,11** |
|  |  | glycylleucine | C02155 | | **3,11** | | | **4,32** | **37,09** | **8,59** |
|  |  | glycylphenylalanine |  | | **2,63** | | | **3,07** | **19,81** | **6,45** |
|  |  | glycyltyrosine |  | | **2,56** | | | **3,45** | **18,39** | **5,32** |
|  |  | glycyltryptophan |  | | **2,36** | | | **2,56** | **41,34** | **16,17** |
|  |  | arginylvaline |  | | 0,88 | | | **3,3** | **39,56** | **12** |
|  |  | aspartylphenylalanine |  | | **4,05** | | | **4,23** | **17,97** | **4,25** |
|  |  | prolylglycine |  | | **0,55** | | | **2,03** | **4,92** | **2,42** |
|  |  | leucylproline |  | | **3,05** | | | **2,28** | **21,73** | **9,52** |
|  |  | aspartylvaline |  | | 0,81 | | | **1,88** | **10,71** | **5,7** |
|  |  | aspartylleucine |  | | **3,66** | | | **4,05** | **16,73** | **4,13** |
|  |  | histidylleucine |  | | **4,25** | | | **5,18** | **55,07** | **10,64** |
|  |  | histidylisoleucine* |  | | 1,04 | | | 0,97 | **12,18** | **12,58** |
|  |  | isoleucylalanine |  | | **3,45** | | | **4,57** | **13,71** | **3** |
|  |  | isoleucylarginine |  | | 1 | | | 1 | **1,55** | **1,55** |
|  |  | isoleucylglutamine |  | | **2,8** | | | **2,12** | **8,89** | **4,19** |
|  |  | isoleucylglycine |  | | **2,13** | | | **3,25** | **26,7** | **8,22** |
|  |  | leucylarginine |  | | 1 | | | 1 | **1,57** | **1,57** |
|  |  | leucylglycine |  | | 1,07 | | | 1,73 | **4,47** | **2,58** |
|  |  | valyllysine |  | | 0,81 | | | **2,66** | **21,95** | **8,24** |
|  | gamma-glutamyl | gamma-glutamylleucine |  | | **6,97** | | | 1,12 | 1,52 | 1,35 |
|  |  | gamma-glutamylisoleucine* |  | | **3,6** | | | 2,14 | 1 | 0,47 |
|  |  | gamma-glutamylcysteine | C00669 | | **5,34** | | | **0,16** | **0,22** | **1,37** |
|  |  | gamma-glutamylglycine |  | | **49,98** | | | 2,18 | **6,39** | **2,94** |
|  |  | gamma-glutamylmethionine |  | | 1 | | | 1 | 1 | 1 |
|  |  | gamma-glutamylphenylalanine |  | | **2,75** | | | 0,73 | 0,73 | 1 |
|  |  | gamma-glutamyltyrosine |  | | **2,62** | | | 1,53 | 1 | 0,65 |
|  |  | gamma-glutamylalanine |  | | **18,95** | | | 1,02 | **1,42** | **1,39** |
|  | Polypeptide | pro-pro-pro |  | | **2,32** | | | **0,6** | 0,88 | **1,47** |
| Carbohydrate | Aminosugars metabolism | glucosamine | C00329 | | 1 | | | **1,13** | **16,77** | **14,88** |
|  |  | N-acetylglucosamine | C00140 | | **3,86** | | | **62,3** | **8,45** | **0,14** |
|  |  | N-acetylglucosamine 6-phosphate | C00357 | | **2,1** | | | **1,89** | **1,85** | 0,98 |
|  |  | erythronate* |  | | 0,99 | | | **2** | **2,49** | **1,24** |
|  | Fructose, mannose, galactose, starch, and sucrose metabolism | fructose | C00095 | | 0,85 | | | **152,52** | **293,59** | **1,92** |
|  |  | isomaltose | C00252 | | **1,71** | | | **1,85** | **4,86** | **2,63** |
|  |  | mannitol | C00392 | | 0,89 | | | **0,05** | **0,33** | **7,02** |
|  |  | mannose-6-phosphate | C00275 | | 1,46 | | | **110,32** | **306,85** | **2,78** |
|  |  | sorbitol | C00794 | | **10,34** | | | **5,39** | **5,04** | 0,94 |
|  |  | trehalose | C01083 | | **4,11** | | | **2,68** | **1,82** | **0,68** |
|  |  | isomaltotriose | C02160 | | **2,84** | | | **2,82** | **0,24** | **0,08** |
|  | Glycolysis, gluconeogenesis, pyruvate metabolism | 2-isopropylmalate | C02504 | | 0,41 | | | **2,9** | **5,56** | **1,92** |
|  |  | glycerate | C00258 | | **0,47** | | | **0,57** | 1,31 | **2,31** |
|  |  | glucose-6-phosphate (G6P) | C00668 | | 1,11 | | | **116,69** | **375,91** | **3,22** |
|  |  | glucose 1-phosphate | C00103 | | 1,22 | | | **5,16** | **15,6** | **3,02** |
|  |  | glucose | C00031 | | **0,04** | | | **2,89** | **92,45** | **32,01** |
|  |  | fructose-6-phosphate | C05345 | | 1 | | | **96,87** | **225,52** | **2,33** |
|  |  | fructose 1-phosphate | C01094 | | 1 | | | **3,04** | 1,88 | **0,62** |
|  |  | Isobar: fructose 1,6-diphosphate, glucose 1,6-diphosphate, myo-inositol 1,4 or 1,3-diphosphate | | | 0,75 | | | **227,72** | **32,48** | **0,14** |
|  |  | 2-phosphoglycerate | | C00631 | | | **0,24** | **0,42** | 0,91 | **2,14** |
|  |  | 3-phosphoglycerate | | C00597 | | | **0,24** | 0,64 | **2,1** | **3,26** |
|  |  | 1,3-dihydroxyacetone | | C00184 | | | 1,33 | 1,09 | **4,09** | **3,75** |
|  |  | phosphoenolpyruvate (PEP) | | C00074 | | | **0,26** | **0,09** | **0,13** | 1,42 |
|  |  | pyruvate | | C00022 | | | **2,31** | **2,27** | **6,82** | **3,01** |
|  |  | lactate | | C00186 | | | **2,36** | 0,95 | **1,35** | **1,42** |
|  |  | 2,3-butanediol | | C03044 | | | **4,42** | **0,51** | 1,22 | **2,39** |
|  | Nucleotide sugars, pentose metabolism | 6-phosphogluconate | | C00345 | | | 1 | **1,34** | **1,47** | 1,09 |
|  |  | arabitol | | C00474 | | | **2,33** | 0,79 | **0,26** | **0,33** |
|  |  | ribitol | | C00474 | | | **4,8** | **1,62** | **1,81** | 1,11 |
|  |  | sedoheptulose-7-phosphate | | C05382 | | | 0,73 | **11,12** | **50,03** | **4,5** |
|  |  | gluconate | | C00257 | | | 0,67 | **26,66** | **10,98** | **0,41** |
|  |  | ribose | | C00121 | | | 1,15 | 1,2 | **9,1** | **7,57** |
|  |  | ribose 5-phosphate | | C00117 | | | 1 | **4,07** | **7,23** | **1,77** |
|  |  | ribulose | | C00309 | | | 1,38 | **7,16** | **3,75** | **0,52** |
|  |  | Isobar: ribulose 5-phosphate, xylulose 5-phosphate | | | | 1 | | **6,06** | **4,19** | 0,69 |
|  |  | UDP-glucose | | C00029 | | | **2,31** | **6,9** | **3,58** | **0,52** |
|  |  | xylulose | | C00310 | | | 1,94 | **7,81** | **16,91** | **2,17** |
| Energy | Krebs cycle | citrate | | C00158 | | | **8,16** | **39,97** | **75,48** | **1,89** |
|  |  | homocitrate | | C01251 | | | **0,59** | **0,29** | **0,54** | **1,85** |
|  |  | alpha-ketoglutarate | | C00026 | | | **8,6** | **12,82** | **7,71** | 0,6 |
|  |  | succinate | | C00042 | | | **1,38** | 0,97 | 1,26 | **1,3** |
|  |  | fumarate | | C00122 | | | **2,68** | **2,44** | **4,19** | **1,72** |
|  |  | mesaconate (methylfumarate) | | C01732 | | | 1,19 | **1,78** | **0,38** | **0,21** |
|  |  | malate | | C00149 | | | 1,13 | **4,06** | **9,07** | **2,24** |
|  | Oxidative phosphorylation | acetylphosphate | | C00227 | | | **1,61** | 1,18 | **1,31** | 1,11 |
|  |  | phosphate | | C00009 | | | 0,82 | **0,62** | 0,96 | **1,56** |
|  |  | pyrophosphate (PPi) | | C00013 | | | **1,8** | 0,93 | **0,04** | **0,04** |
| Lipid | Essential fatty acid | linoleate (18:2n6) | | C01595 | | | **2,8** | **2,46** | **2,43** | 0,99 |
|  |  | linolenate [alpha or gamma; (18:3n3 or 6)] | | C06427 | | | **2,1** | **1,77** | **2,11** | 1,19 |
|  | Medium chain fatty acid | caproate (6:0) | | C01585 | | | 0,63 | 0,39 | 1,34 | **3,46** |
|  |  | heptanoate (7:0) | | C17714 | | | 0,73 | 0,67 | 0,83 | 1,25 |
|  |  | caprylate (8:0) | | C06423 | | | **0,32** | 0,81 | 0,93 | 1,15 |
|  |  | caprate (10:0) | | C01571 | | | 0,79 | **1,53** | 0,77 | **0,5** |
|  | Long chain fatty acid | myristate (14:0) | | C06424 | | | 0,79 | **2,01** | 1,38 | **0,69** |
|  |  | myristoleate (14:1n5) | | C08322 | | | **0,47** | **5,7** | 1,66 | **0,29** |
|  |  | pentadecanoate (15:0) | | C16537 | | | 1,05 | **4,31** | 1,4 | **0,32** |
|  |  | palmitate (16:0) | | C00249 | | | 1,05 | **2,11** | 1,47 | **0,7** |
|  |  | palmitoleate (16:1n7) | | C08362 | | | **0,48** | **3,34** | **2,02** | **0,6** |
|  |  | 10-heptadecenoate (17:1n7) | |  | | | 1,04 | **3,41** | **2,21** | **0,65** |
|  |  | stearate (18:0) | | C01530 | | | 1,27 | **1,9** | **1,78** | 0,94 |
|  |  | oleate (18:1n9) | | C00712 | | | 0,99 | **4,02** | **2,25** | **0,56** |
|  |  | cis-vaccenate (18:1n7) | | C08367 | | | **1,67** | **2,02** | **1,9** | 0,94 |
|  |  | conjugated linoleate (18:2n7; 9Z,11E) | | C04056 | | | **2,5** | **2,15** | **1,84** | 0,85 |
|  |  | eicosenoate (20:1n9 or 11) | |  | | | **1,63** | **1,49** | **2,2** | 1,47 |
|  | Fatty acid, monohydroxy | 4-hydroxybutyrate (GHB) | | C00989 | | | 2,04 | **16,28** | **5,47** | **0,34** |
|  |  | 2-hydroxypalmitate | |  | | | **2,15** | **17,36** | **12,14** | **0,7** |
|  | Fatty acid, dicarboxylate | 2-hydroxyglutarate | | C02630 | | | 0,97 | **2,54** | **5,68** | **2,23** |
|  | Fatty acid metabolism | isovalerate | | C08262 | | | 1 | 1 | 1 | 1 |
|  | Glycerolipid metabolism | ethanolamine | | C00189 | | | 1,54 | **13,27** | **2,84** | **0,21** |
|  |  | phosphoethanolamine | | C00346 | | | 1,25 | **3,11** | **2,5** | 0,8 |
|  |  | glycerophosphoethanolamine | | C01233 | | | **2,06** | **5,18** | 2,3 | **0,44** |
|  |  | glycerol | | C00116 | | | 1,08 | 1,26 | **1,7** | **1,35** |
|  |  | glycerol 3-phosphate (G3P) | | C00093 | | | **3,28** | **2,21** | 1,12 | **0,51** |
|  |  | glycerophosphorylcholine (GPC) | | C00670 | | | **3,21** | **2,87** | **3,25** | 1,13 |
|  |  | cytidine 5'-diphosphocholine | |  | | | **1,55** | **0,64** | **0,46** | **0,72** |
|  | Inositol metabolism | myo-inositol | | C00137 | | | 1,07 | **1,46** | 0,77 | **0,53** |
|  |  | inositol 1-phosphate (I1P) | |  | | | **0,7** | **2,2** | **1,83** | 0,83 |
|  | Ketone bodies | 3-hydroxybutyrate (BHBA) | | C01089 | | | 1,07 | 0,91 | 0,88 | 0,96 |
|  |  | 1,2-propanediol | | C00717,C02912,C00583,C01506,C02917 | | | **3,15** | **1,83** | 1,27 | **0,69** |
|  | Lysolipid | 1-myristoylglycerophosphoethanolamine | | | | **0,34** | | **0,49** | 0,79 | 1,62 |
|  |  | 1-palmitoylglycerophosphoethanolamine | |  | | | **1,95** | **0,25** | 0,98 | **3,88** |
|  |  | 2-palmitoylglycerophosphoethanolamine* | | | | **3,69** | | **0,53** | 0,72 | 1,38 |
|  |  | 1-palmitoleoylglycerophosphoethanolamine* | | | | **2,53** | | **0,21** | 1,25 | **6,07** |
|  |  | 2-palmitoleoylglycerophosphoethanolamine* | | | | **1,54** | | **0,28** | 1,18 | **4,2** |
|  |  | 1-stearoylglycerophosphoethanolamine | | | | 1,1 | | 1,11 | 1,29 | 1,16 |
|  |  | 1-oleoylglycerophosphoethanolamine | |  | | | **3,53** | **0,14** | 0,54 | **3,97** |
|  |  | 2-oleoylglycerophosphoethanolamine* | | | | **1,95** | | **0,12** | 1,01 | **8,34** |
|  |  | 1-linoleoylglycerophosphoethanolamine* | | | | **2,86** | | **0,44** | 1,43 | 3,23 |
|  |  | 2-linoleoylglycerophosphoethanolamine* | |  | | | **1,96** | 1,02 | 2,65 | 2,61 |
|  |  | 1-myristoylglycerophosphocholine | |  | | | **4,04** | 0,78 | **3,89** | **4,96** |
|  |  | 1-palmitoylglycerophosphocholine | |  | | | **8,51** | 0,67 | **5,13** | **7,66** |
|  |  | 2-palmitoylglycerophosphocholine* | |  | | | **10,33** | 1,02 | **2,61** | **2,57** |
|  |  | 1-palmitoleoylglycerophosphocholine* | | | | **2,31** | | **0,42** | **3** | **7,22** |
|  |  | 2-palmitoleoylglycerophosphocholine* | | | | **1,92** | | **0,29** | **2,42** | **8,44** |
|  |  | 1-stearoylglycerophosphocholine | |  | | | **22,85** | 0,82 | **5,4** | **6,58** |
|  |  | 1-oleoylglycerophosphocholine | |  | | | **9,8** | **0,11** | 1,19 | **10,83** |
|  |  | 2-oleoylglycerophosphocholine* | |  | | | **4,87** | **0,17** | 1,41 | **8,08** |
|  |  | 1-linoleoylglycerophosphocholine | | C04100 | | | **16,58** | **0,73** | **1,6** | **2,2** |
|  |  | 2-linoleoylglycerophosphocholine* | |  | | | **5,28** | 1 | 1,01 | 1,01 |
|  |  | 1-palmitoylglycerophosphoinositol* | |  | | | **4,14** | **4,87** | **4,63** | 0,95 |
|  |  | 1-palmitoleoylglycerophosphoinositol* | |  | | | 0,48 | 1,57 | 1,36 | 0,87 |
|  |  | 2-palmitoleoylglycerophosphoinositol* | |  | | | **0,41** | 0,55 | 2,47 | **4,5** |
|  |  | 1-stearoylglycerophosphoinositol | |  | | | **1,96** | **3,03** | **3,48** | 1,15 |
|  |  | 1-oleoylglycerophosphoinositol* | |  | | | 1,68 | **0,3** | 1 | **3,32** |
|  |  | 2-oleoylglycerophosphoinositol* | |  | | | 1,43 | **0,18** | **2,41** | **13,7** |
|  |  | 1-oleoylglycerophosphoserine | |  | | | **1,86** | 0,5 | 0,61 | 1,22 |
|  |  | 2-oleoylglycerophosphoserine* | |  | | | **1,59** | **0,21** | 1,25 | **6,02** |
|  | Monoacylglycerol | 1-palmitoylglycerol (1-monopalmitin) | |  | | | 1,33 | **3,81** | **3,05** | 0,8 |
|  | Diacylglycerol | 1,3-dipalmitoylglycerol | |  | | | **0,34** | **5,13** | 1,8 | **0,35** |
|  | Sphingolipid | sphinganine | | C00836 | | | **16,91** | **2,63** | **18,44** | **7,01** |
|  |  | phytosphingosine | | C12144 | | | **11,12** | **2,72** | **5,82** | **2,15** |
|  | Mevalonate metabolism | 3-hydroxy-3-methylglutarate | | C03761 | | | **2,68** | 0,8 | 0,8 | 1 |
|  |  | mevalonate | | C02104 | | | 0,89 | 0,72 | 0,34 | **0,48** |
|  | Sterol/Steroid | squalene | | C00751 | | | **1,78** | **1,74** | **3,45** | **1,98** |
|  |  | lanosterol | | C01724 | | | **1,98** | **0,58** | 1,24 | **2,15** |
|  |  | ergosterol | | C01694 | | | **0,56** | **1,49** | **2,08** | **1,4** |
| Nucleotide | Purine metabolism, (hypo)xanthine/inosine containing | xanthine | | C00385 | | | 0,85 | **1,57** | **1,46** | 0,93 |
|  |  | xanthosine | | C01762 | | | 1 | 1 | 1 | 1 |
|  |  | hypoxanthine | | C00262 | | | **1,38** | **0,52** | **2,43** | **4,68** |
|  |  | inosine | |  | | | **1,41** | **3,43** | **3,05** | 0,89 |
|  | Purine metabolism, adenine containing | adenine | | C00147 | | | **4,48** | 0,96 | **7,87** | **8,18** |
|  |  | adenosine | | C00212 | | | **0,11** | **0,3** | **0,58** | **1,9** |
|  |  | 1-methyladenine | | C02216 | | | **6,23** | 1,1 | 1,32 | 1,2 |
|  |  | N1-methyladenosine | | C02494 | | | 0,91 | 0,84 | **2,4** | **2,87** |
|  |  | 2'-deoxyadenosine | | C00559 | | | **2,08** | **2,68** | **1,69** | **0,63** |
|  |  | adenosine 3'-monophosphate (3'-AMP) | | C01367 | | | 0,77 | **2,61** | **6,56** | **2,52** |
|  |  | adenosine 5'-monophosphate (AMP) | | C00020 | | | 0,78 | **0,14** | 1,24 | **8,69** |
|  |  | adenosine 5'-diphosphate (ADP) | | C00008 | | | 0,97 | **3,35** | **3,89** | 1,16 |
|  |  | adenosine-2',3'-cyclic monophosphate | | C02353 | | | **0,54** | 1,01 | **1,9** | **1,87** |
|  |  | adenylosuccinate | | C03794 | | | 1,15 | **0,13** | 1,93 | **15,08** |
|  | Purine metabolism, guanine containing | guanine | | C00242 | | | **0,21** | **0,21** | 0,56 | **2,73** |
|  |  | 7-methylguanine | | C02242 | | | **0,55** | **0,8** | **3,11** | **3,89** |
|  |  | guanosine | | C00387 | | | 0,43 | 0,85 | 1,07 | 1,26 |
|  |  | guanosine 5'- monophosphate (5'-GMP) | |  | | | **0,17** | **0,02** | 0,82 | **35,36** |
|  |  | guanosine-2',3'-cyclic monophosphate | | C06194 | | | **1,63** | 1,35 | **3,34** | **2,48** |
|  |  | 2'-O-methylguanosine | | C04545 | | | **1,8** | 0,64 | **2,65** | **4,13** |
|  |  | N6-carbamoylthreonyladenosine | |  | | | **3,63** | **0,36** | **0,35** | 0,96 |
|  | Pyrimidine metabolism, cytidine containing | cytidine | | C00475 | | | 0,81 | **1,63** | **2,79** | **1,71** |
|  |  | cytidine 5'-monophosphate (5'-CMP) | | C00055 | | | **0,33** | **0,04** | 0,61 | **16,47** |
|  | Pyrimidine metabolism, orotate containing | orotate | | C00295 | | | 0,24 | **0,18** | **3,31** | **18,29** |
|  | Pyrimidine metabolism, thymine containing | thymine | | C00178 | | | **0,66** | 0,79 | **1,39** | **1,75** |
|  |  | thymidine | | C00214 | | | **2,1** | **2,34** | 0,63 | **0,27** |
|  |  | thymidine 5'-monophosphate | | C00364 | | | **1,55** | **0,2** | **0,55** | **2,79** |
|  | Pyrimidine metabolism, uracil containing | uracil | | C00106 | | | 1,09 | 1,27 | 1,45 | 1,14 |
|  |  | uridine | | C00299 | | | **1,56** | 1,24 | **2,84** | **2,3** |
|  |  | pseudouridine | | C02067 | | | 0,9 | **0,76** | 1,09 | **1,45** |
|  |  | uridine monophosphate (5' or 3') | |  | | | **0,49** | **0,03** | 1,11 | **33,96** |
|  | Purine and pyrimidine metabolism | methylphosphate | |  | | | 1,17 | **1,52** | **1,6** | 1,06 |
| Cofactors and vitamins | Hemoglobin and porphyrin metabolism | 5-aminolevulinate | | C00430 | | | **1,77** | 0,73 | 1,19 | **1,62** |
|  | Nicotinate and nicotinamide metabolism | nicotinamide | | C00153 | | | 0,62 | 0,97 | 0,95 | 0,97 |
|  |  | nicotinamide ribonucleotide (NMN) | | C00455 | | | **0,3** | **1,85** | **1,74** | 0,94 |
|  |  | nicotinamide adenine dinucleotide (NAD+) | | C00003 | | | **0,67** | **1,26** | **2,15** | **1,7** |
|  |  | nicotinamide adenine dinucleotide reduced (NADH) | | C00004 | | | 1,23 | **3,75** | **7,12** | 1,9 |
|  |  | quinolinate | | C03722 | | | 1,21 | **0,08** | **0,06** | 0,8 |
|  |  | nicotinate | | C00253 | | | **0,48** | 0,66 | **12,97** | **19,8** |
|  |  | nicotinate ribonucleoside* | |  | | | **1,88** | **3,9** | **31,87** | **8,18** |
|  | Pantothenate and CoA metabolism | pantothenate | | C00864 | | | 1,02 | **9,75** | **7,24** | **0,74** |
|  |  | phosphopantetheine | | C01134 | | | **1,3** | **0,25** | **0,55** | **2,2** |
|  |  | coenzyme A | | C00010 | | | **2,09** | 1 | **2,66** | **2,65** |
|  |  | 3'-dephosphocoenzyme A | | C00882 | | | 1,32 | 1,11 | **2,49** | **2,24** |
|  |  | acetyl CoA | | C00024 | | | 1,58 | 0,73 | **5,13** | **7,02** |
|  |  | 2,3-dihydroxyisovalerate | | C04039 | | | 0,75 | **10,25** | **42,65** | **4,16** |
|  | Pyridoxal metabolism | pyridoxal | | C00250 | | | **1,66** | **2,67** | **1,74** | **0,65** |
|  | Riboflavin metabolism | flavin adenine dinucleotide (FAD) | | C00016 | | | **1,47** | **0,54** | **0,47** | 0,87 |
|  |  | riboflavin (Vitamin B2) | | C00255 | | | 1 | 1 | 1 | 1 |
|  |  | flavin mononucleotide (FMN) | | C00061 | | | 0,79 | 1,22 | 0,75 | **0,62** |
|  | Thiamine metabolism | thiamin (Vitamin B1) | |  | | | 0,65 | **2,08** | **5,6** | **2,69** |
|  |  | thiamin diphosphate | | C00068 | | | **0,6** | 1,17 | 1,21 | 1,04 |
|  | Vitamin B6 metabolism | pyridoxine (Vitamin B6) | | C00314 | | | **1,8** | **1,32** | **1,41** | 1,07 |
| Xenobiotics | Benzoate metabolism | p-aminobenzoate (PABA) | | C00568 | | | 1,02 | 1,2 | **0,57** | **0,48** |
|  | Chemical | glycolate (hydroxyacetate) | | C00160 | | | 1,12 | **1,54** | **4,54** | **2,96** |
|  |  | glycerol 2-phosphate | | C02979,D01488 | | | **2,6** | **2,57** | **2,19** | 0,85 |
|  |  | 2-pyrrolidinone | |  | | | 1,32 | **14,06** | **6,62** | **0,47** |
|  |  | oxamate | | C01444 | | | 1,25 | 1,15 | 0,96 | 0,84 |
|  | Food component/Plant | allose | | C01487 | | | **5,74** | **2,67** | **0,67** | **0,25** |
|  |  | quinate | | C00296 | | | **0,44** | **5,2** | **9,61** | 1,85 |
|  |  | shikimate | | C00493 | | | **3,48** | **6,87** | **0,31** | **0,05** |
|  | Sugar, sugar substitute, starch | trehalose 6-phosphate | | C00689 | | | 1 | **2,48** | **1,17** | **0,47** |
|  |  | erythritol | | C00503 | | | **3,01** | **4,8** | **6,87** | 1,43 |
|  | Bacterial | 3-deoxyoctulosonate | |  | | | 0,96 | 1,3 | 1,13 | 0,87 |
| N/A | N/A | glucosylglycerol | |  | | | 1,12 | 1,18 | **4,59** | **3,89** |
